# Data mining to understand health status preceding traumatic brain injury

**DOI:** 10.1038/s41598-019-41916-5

**Published:** 2019-04-03

**Authors:** Tatyana Mollayeva, Mitchell Sutton, Vincy Chan, Angela Colantonio, Sayantee Jana, Michael Escobar

**Affiliations:** 1 Toronto Rehabilitation Institute-University Health Network, Toronto, Canada; 2 Dalla Lana School of Public Health, University of Toronto, Toronto, Canada; 3 Rehabilitation Sciences Institute, Faculty of Medicine, University of Toronto, Toronto, Canada

## Abstract

The use of precision medicine is poised to increase in complex injuries such as traumatic brain injury (TBI), whose multifaceted comorbidities and personal circumstances create significant challenges in the domains of surveillance, management, and environmental mapping. Population-wide health administrative data remains a rather unexplored, but accessible data source for identifying clinical associations and environmental patterns that could lead to a better understanding of TBIs. However, the amount of data structured and coded by the International Classification of Disease poses a challenge to its successful interpretation. The emerging field of data mining can be instrumental in helping to meet the daunting challenges faced by the TBI community. The report outlines novel areas for data mining relevant to TBI, and offers insight into how the above approach can be applied to solve pressing healthcare problems. Future work should focus on confirmatory analyses, which subsequently can guide precision medicine and preventive frameworks.

## Introduction

With the enormous progress in the consolidation of large clinical datasets and national registries in modern healthcare^1,2^, vast amounts of personal, clinical, and environmental data are increasingly becoming available for research^3^. This presents an opportunity to identify novel associations and complex patterns of patient morbidity, personal circumstances, treatment seeking behaviours, and care over time, promoting scientific advancements in personalized medicine for the most complex disorders and injuries^3–5^.

Traumatic brain injury (TBI), defined as structural and/or physiological disruption of brain function as a result of an external force^6^, is rapidly becoming a major challenge faced by healthcare systems worldwide^7^. When internationally reported numbers are extrapolated, it is estimated that 50–60 million individuals are affected each year, and it is predicted that close to 50% of the world’s population will sustain a TBI in their lifetime^8^. The clinical view of TBI has shifted in the last decade from that of an injury event, to a chronic disorder with lifelong effects on both morbidity and mortality^9^, which expedites development of new clinical entities (comorbidities) over time, bringing complexity to its management^10^. Recently, TBI has been recognized as a consequence of multiple comorbid disorders which can potentiate or modify the risks associated with falls or adverse behaviors (e.g., assault and domestic abuse), including but not limited to depressive and substance-use disorders, epilepsy, vascular disease, psychosis, and medication effects^11–20^. Adding to this complexity, any single known adverse determinant of health (e.g. advanced age, socioeconomic deprivation, and gender inequality)^21^ can be implicated in the development of multiple comorbid disorders, thus increasing vulnerability to injury as a result of decreased physical and cognitive reserves^22^.

The World Health Organization (WHO) identified the prevention of injuries as a priority given the projected 40% increase in global deaths due to injury between 2002 and 2030^23^. Likewise, the United States Congress, through Public Law 110–252^24^, highlighted injury surveillance as a federal priority given drastic increase in emergency department visits and hospitalizations for TBI over the past decades^25^. Primary prevention efforts are those designed to prevent the initial injury^26^. Although several studies describe medical and environmental factors associated with an increased risk of TBI including low socioeconomic status, youngest and oldest age groups, male sex, and place of residence^27–30^, these studies are not population-based, and their results may impose a ceiling effect based on the research hypotheses, selected populations with a wide variety of providers and specialists, as well as researcher knowledge and expertise. Based on the fundamental assumption that causal factors of TBI can be identified through the systematic examination of different populations, or of subgroups within populations^31^, successful prevention of TBI is theoretically possible with comprehensive concurrent evaluation of personal, clinical, and environmental contexts in the period prior to TBI^32^ in populations and population subgroups.

Here we describe exploratory research utilizing a data mining non-hypothesis driven approach used in genomics^33,34^ applied to a population which used emergency and acute care resources following TBI, and comparing them to a matched population (individually matched based on age, sex, income level, and place of residence) who used emergency and acute care resources for reasons other than TBI. The focus of this research is not only the data mining methodology, but also the results obtained through data mining approach sequencing more than 70,000 diagnosis codes within the International Statistical Classification of Diseases and Related Health Problems, Tenth Revision (ICD-10) codes within the five years preceding TBI event.

## Methods

### Data sources

Residents of Ontario, Canada’s largest province, have universal public health insurance covering all medically necessary services. The Institute for Clinical Evaluative Sciences (ICES)^35^ houses high quality health administrative data on a wide variety of publicly funded services provided to residents, including but not limited to individual-level information on emergency departments (ED) (identified in the National Ambulatory Care Reporting System, NACRS), and acute care visits (identified in the Discharge Abstract Database, DAD), within the province. The NACRS and DAD contain hospital records with diagnoses^35^, among other personal and environmental data, which are indicated by entries under the ICD-10 Canadian Enhancement (ICD-10-CA)^36^.

### Study design and big data

An observational study was conducted using health administrative data of all patients discharged from the ED or acute care in the province between the fiscal years 2007/08 and 2015/16 with a diagnostic code for TBI^37^ (ICD-10- CA codes S02.0, S02.1, S02.3, S02.7, S02.8, S02.9, S04.0, S07.1, and S06). Personal, clinical, and environmental data for each patient were stored at the ICES; data collected five years prior to each TBI event was extracted for each patient and used in the analysis. A 10% random sample of patients discharged from the ED or acute care during the same study period for a reason other than TBI, individually matched to TBI patients by age, sex, income level, and place of residence (urban vs. rural), was used as a reference population. The first incident of TBI was chosen as the index date for patients with TBI, whereas, for a reference population the midpoint of the ED or acute care visits was selected. To protect against overfitting and for internal validation, the matched dataset was split into three datasets, i.e., training, validation, and testing, with an allocation of 50%, 25%, and 25%, respectively^38^.

### Statistical approach

An association analysis was conducted using ICD-10-CA codes among every patient with TBI and matched patients from the reference population. All ICD-10-CA codes across the 10 and 25 diagnoses fields of the NACRS and the DAD, respectively, were converted into 2600 binary variables. This was done by using the first 3 characters in the ICD-10 codes. The individual codes are nested in these three-character blocks. This was done for each patient’s visit during the five-year period preceding the first TBI event, with the exception of provisional codes for research and temporary assignments, U98 and U99. Following this, a histogram for the days from index date of hospital visits for all TBI patients was constructed. A peak was observed around the index date with the frequency dropping to a stationary point 30 days before, and after, the index date (Supplementary Figs 1 and 2). The 60-day window, therefore, was determined as a TBI-related window, whereas all ED and acute care visits within five years up to 30 days prior to a TBI event were considered to be the pre-injury phase, and were the focus of this study. A similar procedure was performed for each patient in the reference population sample, with the exception that the midpoint of each patient’s hospital visits was selected as an index date.

The next step involved a matched McNemar test on the training dataset for each of the 2600 ICD-10-CA code variables using multiple testing methods^39^ to determine differences between the two groups (i.e., TBI and the reference population) within the period of five years preceding a TBI or an index date event. The Benjamini-Yekutieli multiple testing method^40^ was used to identify a threshold at which results are considered significant given a set of experimental circumstances, and to obtain a set of codes that were significantly overrepresented in TBI patients compared to matched patients from a reference population (i.e., had an odds ratio (OR) greater than one); the False Discovery Rate^40^ (i.e., an approach not commonly used in public health research, but standard in genomic research)^41^ was controlled at five percent. This set of codes was then re-tested on the validation dataset following the same procedure^42^. Codes found to be significant in both the training and validation datasets then had their OR calculated and reported utilizing the testing dataset. It is important to note that the training dataset was twice as large as the validating or the test dataset and consequently, these last two datasets had less power to observe significant effects.

Further, data dimensionality and codes reduction were examined using a factor analysis technique (i.e., principle components methods)^43^ with the following criteria to determine the number of factors: (i) eigenvalue larger than one; (ii) break-point on the scree plot; (iii) the greatest cumulative proportion of variance accounted for; and (iv) a conditional logistic regression looped through all possible factors covering the largest area under the receiver operating characteristic (ROC) curves using the validation dataset. A code was included in a given factor if its factor loading was greater than or equal to 0.2, with no limitations placed on any code loaded on multiple factors.

The decision regarding how many factors should be retained was supported by a binary form of a factor-based score. Each patient who had any of the ICD-10-CA codes included in the definition of the factor (based on the criteria above) obtained a score of one; otherwise, a score of zero was assigned. These binary factor-based scores were applied to the testing dataset and were used to calculate ORs and 95% confidence intervals from a looped conditional logistic regression model on the association between each factor and TBI.

Finally, to visualize the results, word cloud figures were generated for the frequencies and ORs of the factors, where the size of the words indicated the different magnitudes of these values.

All statistical analyses were conducted using SAS software (version 9.410, SAS Inc., Cary, NC) and R (version 3.4.1.11), R Foundation for Statistical Computing; www.r-project.org). Figures were created using R with the Wordcloud package.

## Results

Among the overall Ontario population of between 12.9 and 14 million in 2008 and 2016^44^, respectively, 239,103 unique patients had their first TBI-related visit in either an ED or acute care setting between the fiscal years 2007/08 and 2015/16. Each patient with TBI was matched to a patient from the 10% random sample of patients entering an ED or acute care setting for any reason other than TBI; 4,100 (1.7%) patients were left unmatched and were excluded from analysis; the final cohort consisted of 235,003 patients. This sample was randomly split into training (50%; n = 117,689), validation (25%; n = 58,798), and testing (25%; n = 58,516) datasets. Frequencies, outputs, and measurements were presented on the testing dataset (we refer the reader to the methods section for specifics).

Of the 58,516 patients (and matched reference patients), 57% were males and 62% were 40 years of age or younger when they had their first TBI. In 88% of patients, TBI was cited as the main diagnosis for their ED or acute care visit. Severity of injury was not established in the data files of 64% of the patients, among them, 92% were coded as concussion without a specified length of unconsciousness (ICD-10-CA code S06.0). Accidents accounted for 92% of TBIs and assaults for 7%. Twenty-five percent of all injuries were sports-related, and 10% were related to motor vehicle accidents. Most injuries were sustained as a result of falls (45%) or from being struck by an object or person (36%). During the studied period, patients with TBI had more than twice the average number of hospital visits (emergency or acute) than those in the reference population (4.3 vs. 2.0) (Table 1).

**Table 1:** Characteristics of patients with first traumatic brain injury-related visit in the emergency department or acute care and matched reference patients between April 1, 2007 and March 31, 2016.

Variables	Patients with TBI (N = 58,516)	Reference patients (N = 58,516)
**Sociodemographic characteristics**		
Sex,		
Male	33,379 (57)	33,379 (57)
Female	25,137 (43)	25,137 (43)
*Age at injury (years)*	36.23 (25.33)	36.24 (25.32)
*Income quintile*		
Q1 (lowest)	11,465 (20)	11,465 (20)
Q2	11,540 (20)	11,540 (20)
Q3	11,494 (20)	11,494 (20)
Q4	12,182 (20)	12,182 (20)
Q5 (highest)	11,835 (20)	11,835(20)
*Rurality*	9,084 (16)	9,084 (16)
**TBI-related characteristics**		
*TBI main diagnosis*	51,705 (88)	NA
*Injury severity*		
Unspecified	37,466 (64)	NA
Mild	8,259(14)	NA
Moderate	2,068 (4)	NA
Severe	10,723 (18)	NA
*Type of first healthcare entry*		
Emergency Department	48,142 (82)	NA
Acute Care	4,147 (7)	NA
Emergency & Acute*	6,227 (11)	NA
*Context of Injury*		
Sports injury	14,472 (25)	NA
Assault	4,355 (7)	NA
*Mechanism of injury*		
Falls	26,480 (45)	NA
Motor vehicle accidents	5,808 (10)	NA
Struck by/against	20,845 (36)	NA
Other	5,266 (9)	NA
*Number of hospital visits*	4.25 (7.70)	1.97 (3.44)

Definition of abbreviations: Q = quantile; NA = not applicable; TBI = traumatic brain injury; SD = standard deviation.

Data are given as mean (standard deviation) or n (%). *A patient had a transfer to either location on the same day.

Matched McNemar tests were performed for all 2,600 ICD-10- CA binary variables on the training dataset. The Benjamini-Yekutieli multiple testing method identified 775 significant associations, of which 684 (88.3%) had an OR greater than one. These 684 codes were re-tested on the validation dataset, and 582 of them (85.1%) were internally validated.

Factor analysis was performed on the training dataset on 582 of the ICD-10- CA codes. Of the 582 codes included in the analysis, 329 (56.5%) individual codes met the factor loading cut-off of 0.2. Supplementary Tables 1 and 2 present the individual frequencies, ORs, and factor loadings of codes that met the factor analysis cut-off and codes that did not meet the cut-off, respectively.

Using the break-points on the scree plots and the interpretability, 43 factors were selected. The scree plots are presented in Supplementary Figs 3 and 4 with the values in Supplementary Table 3. Table 2 presents the descriptions, frequencies, ORs, and ICD-10 codes included for each of the 43 factors.

**Table 2:** Matrix of factor analyses with ICD-10-CA codes loading, disease category and effect (OR and 95% CI).

Factor number	Description	Disease category	ICD-10-CA codes	Frequency in cohorts		OR [95% CI]
				TBI	Reference	
Factor 1	Cardiovascular disorders and other	Cardiology	I25, I21, R94, Z95, I20, E78, I24, I10, I50, I48, I35, R07, E11	9,818 (16.8)	5,434 (9.3)	2.21 [2.12, 2.3]
Factor 2	Mental health disorders; functional inquiry	Psychiatry	R45, F32, F43, F60, Z63, F91, Z91, X78, F31, F41, F33, F34, F90, X61, F61, X69, F39, Z59, F69, X60, F19, Z60, T43, F12, Z56, Z65, T65	4,941 (8.4)	1,828 (3.1)	2.89 [2.74, 3.06]
Factor 3	Disorders of elderly and medical issues	Geriatrics	Z75, R29, S72, F03, R64, G20, F05, R41, W19, W05, W01, R26, W18, F02, M81, Z50, I10, N39, Z74, W06, E87, Z73, R53	14,071 (24.0)	6,446 (11.0)	2.82 [2.72, 2.92]
Factor 4	Orthopedic injuries and other	Trauma	S62, S69, W22, S60, S63, S52, W21, Z47, W51, W02, W23, S50, W19, S59, V18, X59, W18, W01, S90, X50, W50	22,429 (38.3)	8,275 (14.1)	4.10 [3.97, 4.23]
Factor 5	Disorders of renal function and therapy	Nephrology	N18, Z49, Z99, I12, N08, N19, T82, N17	1,160 (2.0)	535 (0.9)	2.29 [2.06, 2.55]
Factor 6	Skin/soft tissue lesions, vascular/lymphatic pathology, back pain	Dermatology Osteopathy/ Orthopedy	L03, Z51, M79, L02, M25, M54, R22, L08, Z09, I80, R07, Z48, Z76, L97	15,166 (25.9)	8,237 (14.1)	2.21 [2.14. 2.28]
Factor 7	Burns	Environmental exposures	T31, T23, X10, T22, X12, T21, T24, X15, T20, T25, X09, X19, X11, X08	661 (1.1)	262 (0.4)	2.55 [2.21, 2.95]
Factor 8	Respiratory infections of upper airway, ear, nose	Otolaryngology	H66, J06, J02, R50, H92, B34, R21, R05, J05, J03, H60, H10, J20	13,054 (22.3)	7,749 (13.2)	2.04 [1.97, 2.11]
Factor 9	Liver disorders and other	Gastroenterology	K74, K72, R18, I85, K76, K70, B18, D61	417 (0.7)	132 (0.2)	3.21 [2.63. 3.91]
Factor 10	Pulmonary, abdominal and other emergencies	Emergency medicine	A41, J17, J96, R57, N17, B95, B96, E87, L89, F05, U82, J15, Z75, J90, J69, A49	3,237 (5.5)	1,766 (3.0)	1.99 [1.87, 2.11]
Factor 11	Metabolic disorders and abdominal symptoms	Gastroenterology	K29, D63, D64, K92, Z85, E87, D50, D46, E86, R11, E83, K21, I10, D61, R10, K56, N17, D69, C79	13,138 (22.5)	8,072 (13.8)	1.90 [1.84, 1.97]
Factor 12	Stroke and emergencies involving brain	Neurology	G81, I63, R47, I64, I69, Z75, I61, G45, I67, I10, I62	3,923 (6.7)	2,332 (4.0)	1.92 [1.82, 2.04]
Factor 13	Adverse drug effects of prescribed medications	Pharmacology emergencies	T42, X61, T43, X41, Y11	508 (0.9)	112 (0.2)	4.57 [3.72, 5.61]
Factor 14	Emergencies and adversities due to substance abuse	Toxicology	F19, F11, F14, F10, Z76, Y04, Z72, Z59, F13, Y09	4,503 (7.7)	1,198 (2.0)	4.13 [3.86, 4.42]
Factor15	Diabetes and diabetic consequences	Endocrinology	E14, E11, E10, R73, H36, N08, I79, L97, G63, M86	3,336 (5.7)	1,938 (3.3)	1.86 [1.75, 1.97]
Factor 16	Conditions and symptoms of abdomen and pelvis	Gastroenterology Obstetrics	R10, N83, Z33, N93, Z32, N94, N73, N39, K37, Z71, G43, K35, N12, R11	12,554 (21.5)	7,130 (12.2)	2.04 [1.98, 2.11]
Factor 17	Chronic cardiovascular pathology	Cardiology	Y44, D68, R78, Z92, I48, I50, I80	2,615 (4.5)	1,339 (2.3)	2.20 [2.05, 2.37]
Factor 18	Superficial injuries	Trauma	V43, S20, S30, S19, S10, S39, T00, M54, S40, V89, T14, S49, T09	8,638 (14.8)	3,067 (5.2)	3.20 [3.06, 3.35]
Factor 19	Acute and chronic disorders of airway and lungs	Infectious diseases and respirology	R06, J45, J18, R05, J44, J98, J06, J40, J20, R07	13,938 (23.8)	7,653 (13.1)	2.14 [2.07, 2.21]
Factor 20	Alcohol-related emergencies	Toxicology	T51, X45, Y15, X65, Y90	186 (0.3)	36 (0.1)	5.17 [3.62, 7.38]
Factor 21	Schizophrenia and delusional disorders	Psychiatry	F20, F29, F25, F22, R44, R46, F31, F99, Z59	1,004 (1.7)	334 (0.6)	3.07 [2.71, 3.48]
Factor 22	Poisoning due to narcotics	Pharmacology emergencies	T40, X42, Y12, X62, F11	468 (0.8)	82 (0.1)	5.71 [4.51, 7.22]
Factor 23	Injuries from contact with sharp instruments and machinery	Trauma	S61, W26, W45, W25, S91, W29, W49, W27, S51, Z48, W31, W23	7,528 (12.9)	3,301 (5.6)	2.54 [2.43, 2.66]
Factor 24	Poisoning due to hormones, cardiovascular drugs and other	Pharmacology emergencies	X44, T50, X64, Y14, T45, T38, T46, T49	420 (0.7)	159 (0.3)	2.66 [2.22, 3.2]
Factor25	Poisoning by pain killers and anti-inflammatory drugs	Pharmacology emergencies	T39, X60, X40, Y10	295 (0.5)	77 (0.1)	3.83 [2.98, 4.92]
Factor 26	Epilepsy, seizures, brain lesions and other	Neurology	G40, R56, G41, Y46, F44, D43, G93, C71	1,121 (1.9)	365 (0.6)	3.11 [2.76, 3.51]
Factor 27	Overexertion and injuries to the lower limb	Trauma	X50, S93, S99, X59, S83, S90, S82, T78, X58	12,053 (20.6)	4,790 (8.2)	3.02 [2.91, 3.14]
Factor 28	Genitourinary disorders, disorders of prostate, other	Nephrology	T83, R33, Z46, R31, Y84, N20, N39, N23, N30, B96	5,379 (9.2)	3,436 (5.9)	1.67 [1.59, 1.75]
Factor 29	Alzheimer’s diseases and dementia	Neurology	G30, F00, F03	530 (0.9)	148 (0.3)	3.81 [3.15, 4.6]
Factor 30	Foreign body in eye, airway and other	Emergency medicine	W44, T15, T18, T17, T16, H18	1,682 (2.9)	624 (1.1)	2.78 [2.53, 3.05]
Factor 31	Complications of medical procedures	Emergency medicine	Y83, T81, Y84, T82	2,600 (4.4)	1,533 (2.6)	1.76 [1.65, 1.88]
Factor32	Exposure to heat and light	Environmental exposures	X30, T67	66 (0.1)	26 (0.0)	2.54 [1.61, 4.0]
Factor 33	Exposure to cold/hypothermia	Environmental exposures	X31, T35, T68	82 (0.1)	21 (0.0)	3.90 [2.42, 6.31]
Factor 34	Bee, wasp and hornet stings	Environmental exposures	T63, X23	349 (0.6)	174 (0.3)	2.01 [1.68, 2.41]
Factor 35	Viral conjunctivitis	Infectious diseases	B30, H13	84 (0.1)	41 (0.1)	2.05 [1.41, 2.98]
Factor 36	Assault and intentional injury	Trauma	X99, S21, S11, S41, T01, S27, X78, S31, S51	1,266 (2.2)	334 (0.6)	3.88 [3.43, 4.38]
Factor 37	Adverse reactions to antibiotics and other drugs	Pharmacology emergencies	Y40, L27, T88, T78, Y57, R21, L50, X58	3,851 (6.6)	1,870 (3.2)	2.15 [2.03, 2.28]
Factor 38	Adult and child abuse and sexual assault	Trauma	T74, Y05, Y07	176 (0.3)	34 (0.1)	5.44 [3.73, 7.93]
Factor 39	Burn and chemical poisoning	Environmental exposures	T26, W89, X49, X08, T54, T65	436 (0.7)	127 (0.2)	3.45 [2.83, 4.21]
Factor 40	Infections of reproductory organs and other	Gynecology	N77, B37, A60, N76	571 (1.0)	273 (0.5)	2.10 [1.82, 2.43]
Factor 41	Poisoning due to drugs acting on autonomic nervous system	Pharmacology emergencies	X63, T44	33 (0.1)	13 (0.0)	2.54 [1.34, 4.82]
Factor 42	Toxic effect of gases, fumes and vapors	Environmental exposures	T59, X47, X09	159 (0.3)	46 (0.1)	3.46 [2.49, 4.8]
Factor 43	Exposure to electrical current	Environmental exposures	W87, T75	64 (0.1)	19 (0.0)	3.37 [2.02, 5.62]

Definition of abbreviations: CI = confidence interval; OR = odds ratio; TBI = traumatic brain injury.

Data are given as n (%) in both TBI and the reference patients’ group. All OR’s p-values are less than 0.0001 except for Factor 35 and Factor 41 (p = 0.0002 and p = 0.0044, respectively).

**Figure 1:**
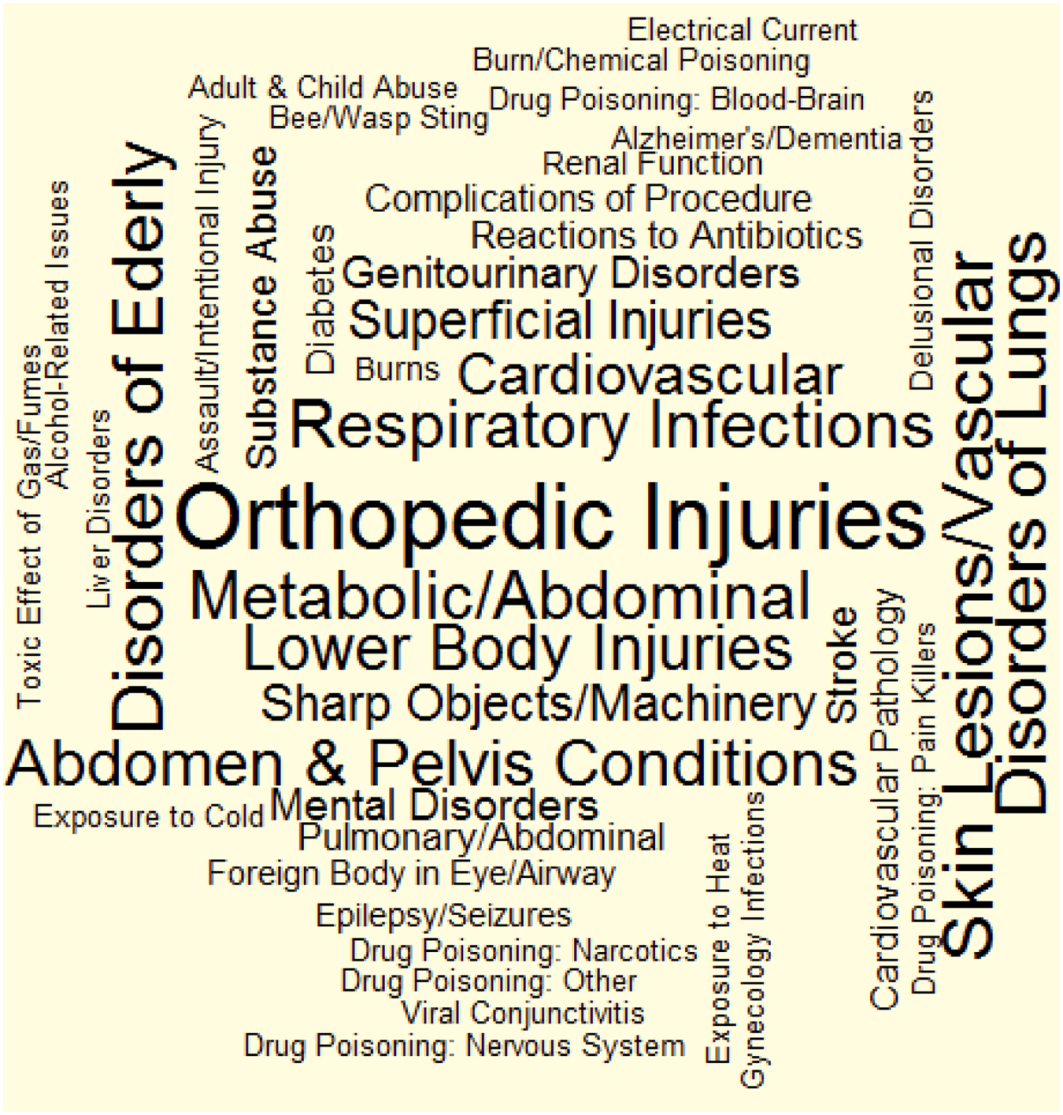
Wordcloud of factors, by frequency. Font size of each factor is proportional to its frequency of occurring preceding the index injury date in individuals with TBI.

When factors were sorted by frequency in the TBI population, those related to general trauma (Factors 4, 27, and 18), dermatology (Factor 6), geriatrics (Factor 3), respirology (Factor 19), otolaryngology (Factor 8), gastroenterology (Factors 11 and 16), and cardiology (Factors 1 and 17) had a high rate of occurrence, while factors related to environmental exposure (Factors 43, 32, 33, 42, and 34), pharmacology emergencies (Factors 41 and 25), abuse trauma (Factor 38), toxicology (Factor 20), and infectious diseases (Factor 35) occur less frequently. For a visual representation of these frequencies, we refer the reader to the Wordcloud in Fig. 1.

**Figure 2:**
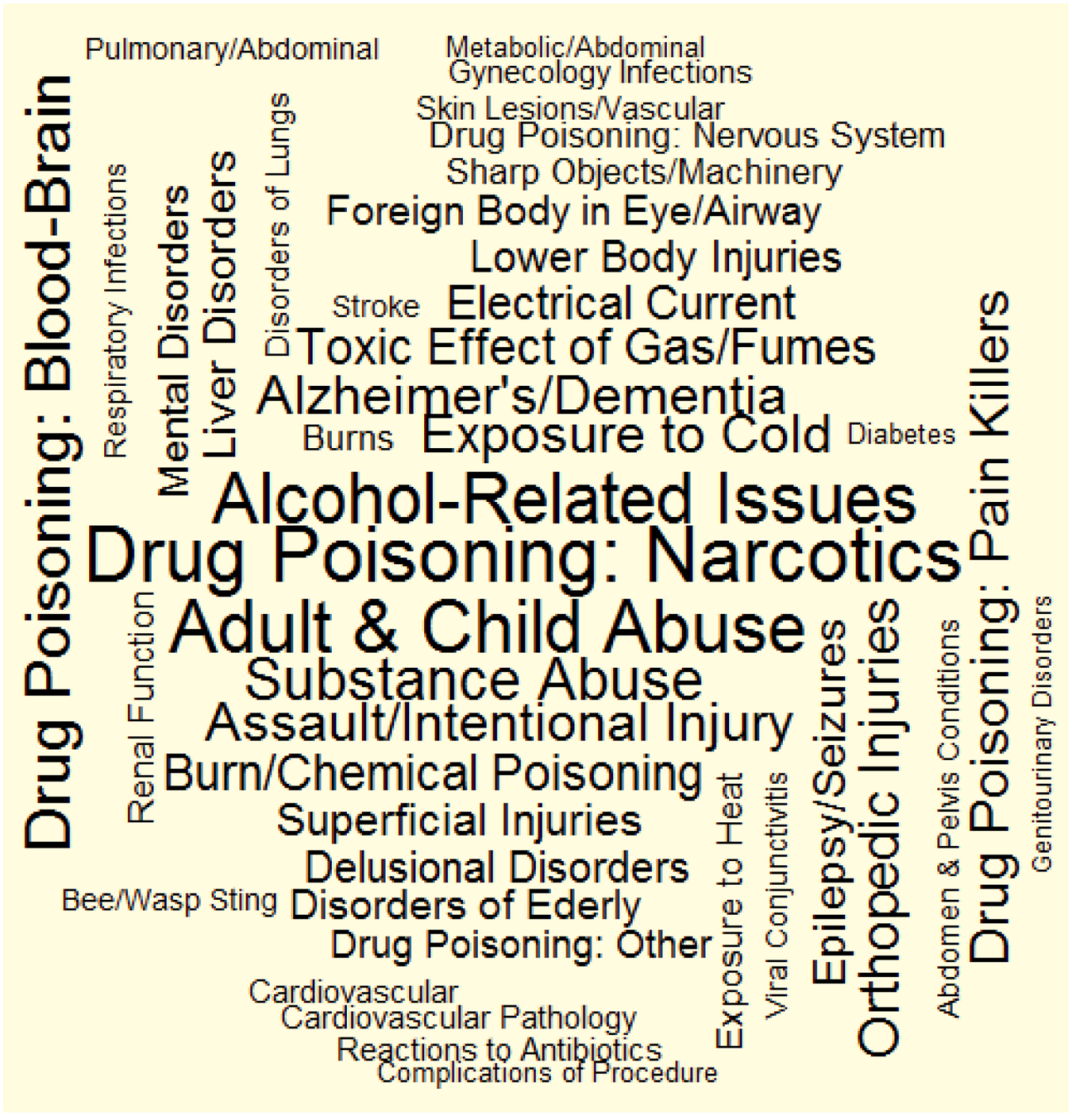
Wordcloud of factors, by magnitude of effect size. Font size of each factor is proportional to its odds of occurring preceding the index injury date in individuals with TBI relative to the reference population of similar age, sex, income level, and place of residence. In other words, people with pharmacology emergencies, alcohol-related issues, exposed to abuse or assault, or environmental hazards and other factors listed in the figure, are more likely to be present in patients visiting emergency department or acute care due to TBI than due to any other reason but TBI.

When factors were sorted by the magnitude of the effect size (OR), factors related to pharmacology-related emergencies (Factors 22, 13, and 25), abuse (Factors 38 and 36), toxicology (Factors 20 and 14), general trauma (Factor 4), environmental exposures (Factor 33), and Alzheimer’s/dementia (Factor 29) have a stronger association with TBI, while factors related to nephrology (Factor 28), emergency medicine (Factors 31 and 10), endocrinology (Factor 15), gastroenterology (Factors 11 and 16), stroke (Factor 12), otolaryngology (Factor 8), and infectious diseases (Factor 35) have a weaker association. For a visual representation of these ORs, please see the Wordcloud in Fig. 2.

Supplementary Table 3 provides details of the demographics (i.e., age, sex, income, and rurality distributions) of each of the 43 factors in the TBI, and reference patient populations.

## Discussion

As data mining is known to be useful for clinical data^45^, the focus is naturally turned to exploring health administrative data for improving the surveillance, management, and environmental mapping of complex injuries and disorders. Rich and structured patient data encoded in ICD-10 diagnostic fields significantly expand researchers’ ability to phenotype the profiles of patients at the pre-injury phase, both within the specific clinical pathology (comorbidity), as well as for environmental exposures and circumstances. Combining ICD-10 codes and personal characteristics of patients in the timeframe preceding TBI creates enormous opportunities for not only precision medicine^46^, but also for injury prevention^47^.

The data mining procedure applied here represents a novel non-hypothesis driven approach for dealing with complex medical issues and big data simultaneously, when manual inspection of valuable clinical and non-clinical information from each patient individually and then the population as a whole, would be an otherwise impossible task. We showed how administrative healthcare information can be used in categorizing multidimensional comorbidities, how multiple comorbidities load on individual factors, and how to perform factor reductions to maximize the cumulative percentage of explained variances, and enhance the clinical interpretability of results. Finally, the data mining approach developed allowed not only the validation of previously known risk factors of TBI, but also shed light on the magnitude of associations that previously received little attention, including those related to exposure to occupational hazards, both chemical (i.e., gases, mineral dusts), physical (i.e., extreme temperatures) and mechanical (i.e., trauma); the long-lasting concerns of assault and child abuse at the population level and their links to TBI, and the adverse effect of medications and drugs in the years preceding TBI. Such novel associations as exposure to toxic gases and fumes, and neurotoxicity of prescription drugs, are extremely important for the future of research and practice pertaining to concussions without a specified length of unconsciousness (S06.0), where there is considerable debate over the clinical definition, neurological signs, and clear epidemiological evidence of probable causation between certain clinical and environmental factors and the injury itself^48^, or where there is a need to differentiate the effects of neurotoxic drugs from those of TBI. When examining ICD-10-CA factors among patients with TBI by rows (Supplementary Table 3), it is evident that multiple medical conditions, which are well-known TBI risk factors^15,18,19,49^, are present within identified factors in the years preceding TBI. Instead of a binary association of a given code (or multiple codes) with a given patient, we presented the significance of the occurrences of the ICD-10 codes and associated factors using their frequency distribution in TBI patients and the reference population matched with age, sex, income level, and place of residence. It was observed that factors composed of cardiovascular and metabolic disorders, orthopaedic injuries, mental health disorders, dementias, and Parkinson’s disease are highly overrepresented in the five-year timeframe preceding an individual’s first TBI, as compared to the reference population. The above factors are known to be implicated in the risk of falls and motor vehicle accidents^50,51^. Overdose of prescription drugs highlighted here also play a role – drugs that cross the blood-brain barrier affect brain functioning and alertness and/or cause postural hypotension, increasing the risk of falls^52,53^. Likewise, pain killers, especially opioid medications, frequently cause opioid induced respiratory depression, a combination of a lowered level of consciousness, decreased respiratory drive, and upper airway obstruction, and are implicated in cerebral hypoxia and falls with or without the loss of consciousness^54,55^. Along with prescribed medications, alcohol abuse is a major risk factor for TBI. In more than half of all patients, new TBI occurred at a time when the patient was intoxicated, while excessive drinking increased the risk of dying from head trauma in 36% of assaults, 41% of falls, and 40% of suicidal circumstances^56^. In line with this evidence, alcohol-related issues, poisoning due to narcotics, and other psychotropic medications, were associated with an increased OR of TBI in the near future, compared to the reference population. Recognition of hazardous situations and exposures such as assaults and self-harm injuries preceding TBI diagnosis, as an opportunity to intervene and prevent TBI, cannot be underestimated^57^. However, identified codes loaded on adult and child abuse factors are novel and point to the complexity of social circumstances surrounding any given individual in such a situation, and the seriousness of the lasting adverse effects, highlighting the value of a multifaceted investigation needed in TBI prevention and post injury management.

Perhaps the most unique finding is the high percentage of men and women of working age who sought care after environmental exposures to gases and fumes, electrical currents, sharp objects, machinery, and the cold in the five years preceding their TBI event. Work-related TBIs have important societal impacts particularly in high-income countries where high-risk industries such as construction, transportation, manufacturing, farming, fishing, forestry, and mining, are active. The WHO considers “environment” to cover physical, chemical, and biological factors external to the individual^58^. Surveillance in occupational settings where workers are exposed to electrical currents, sharp objects and moving machinery, and geographic-specific interventions that focuses on unique hazards have to be controlled by governmental health and safety organizations. Safety interventions expect to be tailored to the particular hazards to which a worker is exposed to, with ongoing laboratory quality control and regular hygiene investigations at the workplace, to protect workers’ health^59^. While it is a tremendous challenge to alter environmental exposures to prevent TBIs, future research may propose ways to regulate work environments for such exposures as a means to reduce injury rates.

Our data mining approach and discussion have some limitations. Our analysis was focused on ICD-10 codes, which have largely unknown diagnostic accuracy, specificity, and sensitivity values. Our factor analysis highlighted that many codes from different categories were loaded on the same factor, and therefore, clinically they should be considered collectively as a single factor rather than as separate factors. This is the case for not only mental health disorder and cardiovascular disease codes, but also codes related to stroke and medical emergencies, infectious diseases and respirology, seizures, epilepsy, and complications of medical procedures, among others. Some of these codes loading on the same factor might represent shared pathophysiological mechanisms of systemic disorders, while others might represent variation in the use of codes among different medical disciplines across the health system in Ontario. Future improvement in methodologies for factors with competing loading effects of ICD codes should be undertaken, to disentangle the complex interplay of person-environment and the healthcare system interaction, to develop a richer understanding of TBI diagnosis, and to untangle the more complex interplay of processes preceding TBI. The complete data presented can be used for hypothesis generation, and not for making any conclusions about causality, and provide deeper insights into the roles of comorbidity, personal circumstances and environmental exposures in regulating the TBI.

To prevent TBI, a complex and often a lifelong disabling injury, it is essential to understand its distribution and patterns, in addition to having extensive knowledge of any clinical disorder, characteristic, or other definable entity, that differentiates TBI from other clinical populations. The findings of this study add to clinical and technological advancement, in providing new techniques for categorizing personal, clinical, and environmental exposure data, and combination of codes in clinically meaningful factors, with enhanced comprehensibility that could aid in future studies of injury prevention. Possible extensions to this work would involve the application of these novel frameworks for detecting factors at the event and post-event phases that could be targeted for secondary and tertiary prevention. With the support of data mining and big data, it is possible to monitor patients’ health and harmful environmental exposures and advance the fields of both precision medicine and injury surveillance. Future statistical and data mining advancements would require improving sensitivity and interpretability of the proposed methodology by validating the described data mining algorithm using data from a patient population across Canada.

### Ethical approval and informed consent

#### Approval

The study protocol was approved by the ethics committees at the clinical (Toronto Rehabilitation Institute-University Health Network) and academic (University of Toronto) institutions.

#### Accordance

All methods were carried out in accordance with the relevant guidelines and regulations.

#### Informed consent

This research utilised de-identified administrative health data with no access to personal information.

## Supplementary information

Data mining to understand health status preceding traumatic brain injury.

## Data Availability

The datasets generated during and/or analysed during the current study are available in the ICES repository, [www.ices.on.ca/DAS < http://www.ices.on.ca/DAS], under accession DAS 2016-257(2018 0970 084 000). Data sharing agreements prohibit ICES from making the datasets publicly available, however access may be granted to those who meet pre-specified criteria for confidential access. The full dataset creation plan and underlying analytic code are available from the authors upon request, understanding that the computer programs may rely upon coding templates or macros that are unique to ICES and are therefore either inaccessible or may require modification.
