# Supplementary material for: Data mining to understand health status preceding traumatic brain injury
Source: Sci Rep. 2019 Apr 3;9:5574. doi: 10.1038/s41598-019-41916-5 (PMC6447542; doi:10.1038/s41598-019-41916-5)
Supplement: Supplementary file 1 — Data mining to understand health status preceding traumatic brain injury. [file 41598_2019_41916_MOESM1_ESM.pdf]

## Online data supplement

**Title:** Data mining to understand health status preceding traumatic brain injury.

**Authors:** Tatyana Mollayeva, Mitchell Sutton, Vincy Chan, Angela Colantonio, Sayantee Jana, Michael Escobar

## Table of contents

Supplementary Figure 1. A histogram of the number of visits to the hospital by the number of days between the visit and first TBI

Supplementary Figure 2. A boxplot of the number of visits to the hospital by the number of days between the visit and first TBI

Supplementary Figure 3. Scree plot of all factors

Supplementary Figure 4. Scree plot of the first 50 factors. Methods used to extract the optimal numbers of factors were Eigenvalues, the plateau of Eigenvalues (i.e. the 'elbow' in the scree plot), and the variance explained

Supplementary Table 1. Factor descriptions. Provides all ICD-10 codes included in each factor, including their descriptions, frequencies (in TBI and reference population), odds ratio (and 95% confidence interval) from multiple testing results, and factor loading score for each factor

Supplementary Table 2. ICD-10 Codes not included in the Factors. Provides all ICD-10 codes not included in any factor (in alphabetical order), including their descriptions, frequencies (in TBI and reference population), and odds ratio (and 95% confidence interval) from multiple testing results

Supplementary Table 3. Scree table. Provides eigenvalues and their differences used in preparation of scree plots, as well as the proportion/cumulative proportion of total variance explained by each factor; used to make a decision on the number of factors to include in the final model

Supplementary Figure 1. A histogram of the number of visits to the hospital by the number of days between the visit and first TBI.

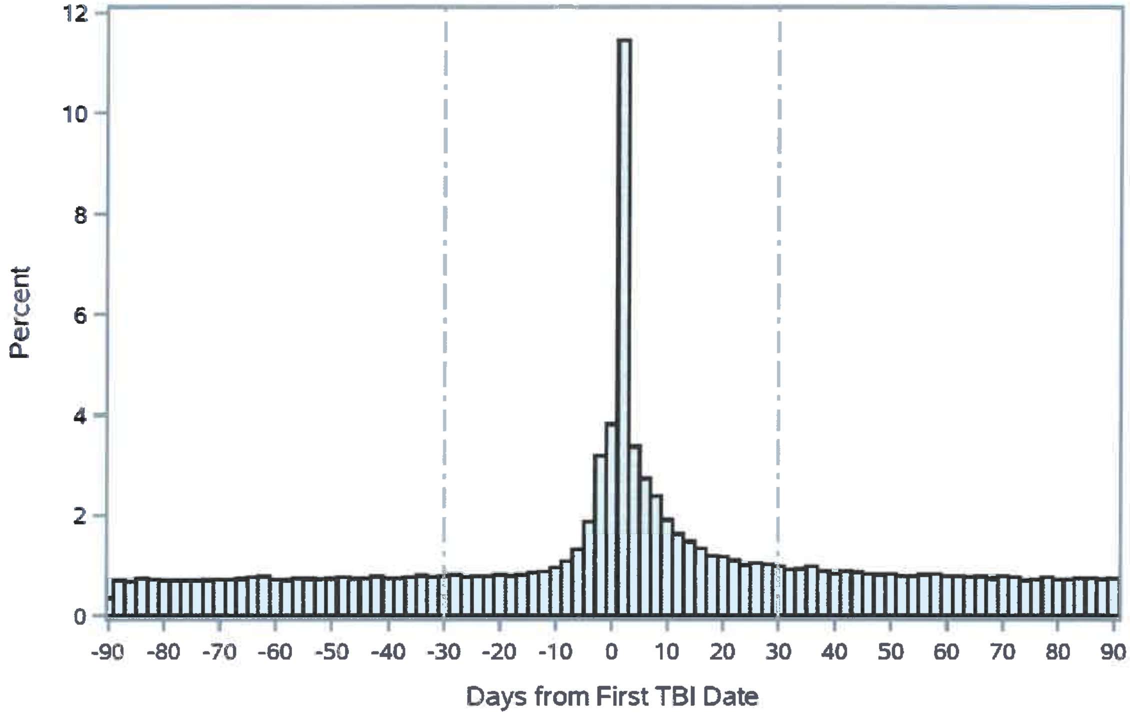


Supplementary Figure 2. A boxplot of the number of visits to the hospital by the number of days between the visit and first TBI.

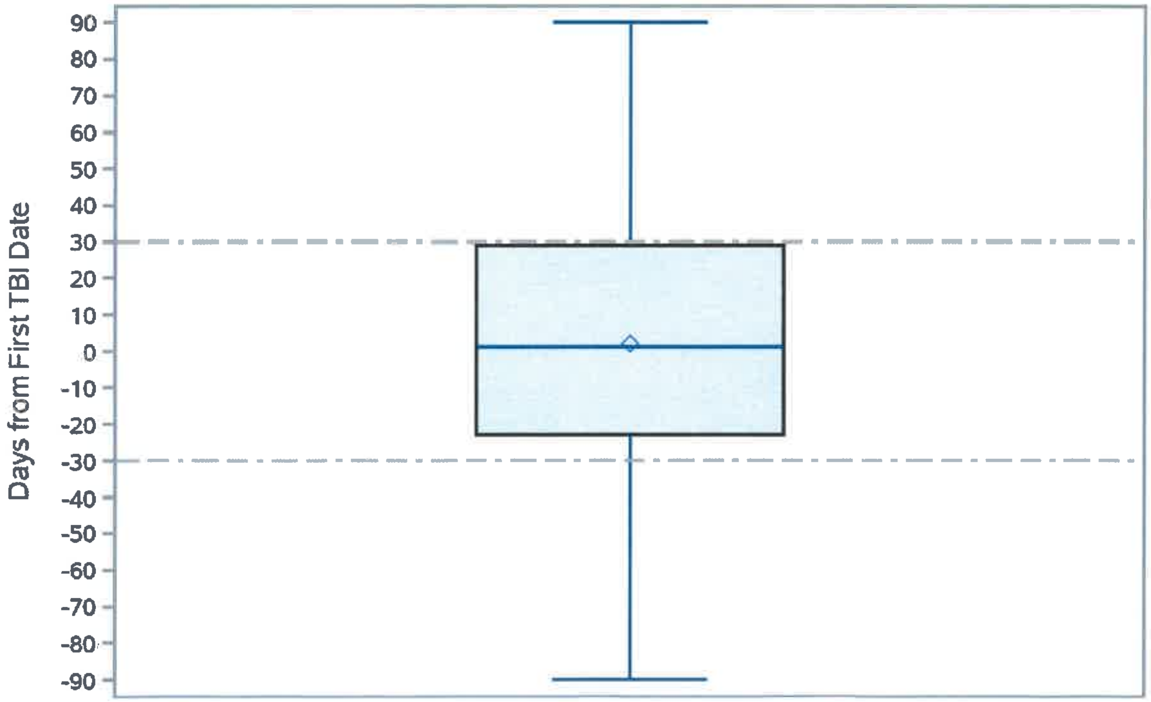

Supplementary Figure 3. Scree plot of all factors.

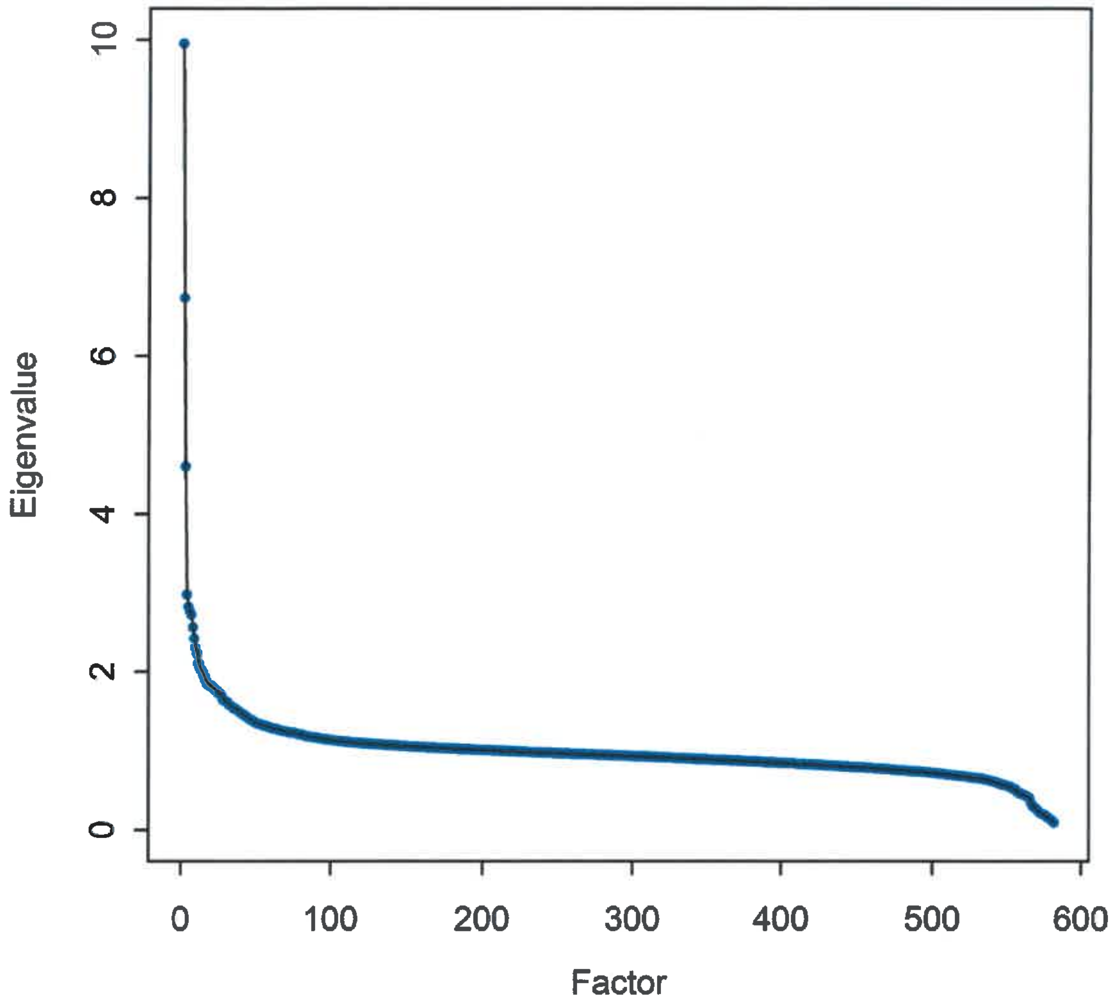

Supplementary Figure 4. Scree plot of the first 50 factors. Methods used to extract the optimal number of factors were Eigenvalues, the plateau of Eigenvalues (i.e. the 'elbow' in the scree plot), and the variance explained.

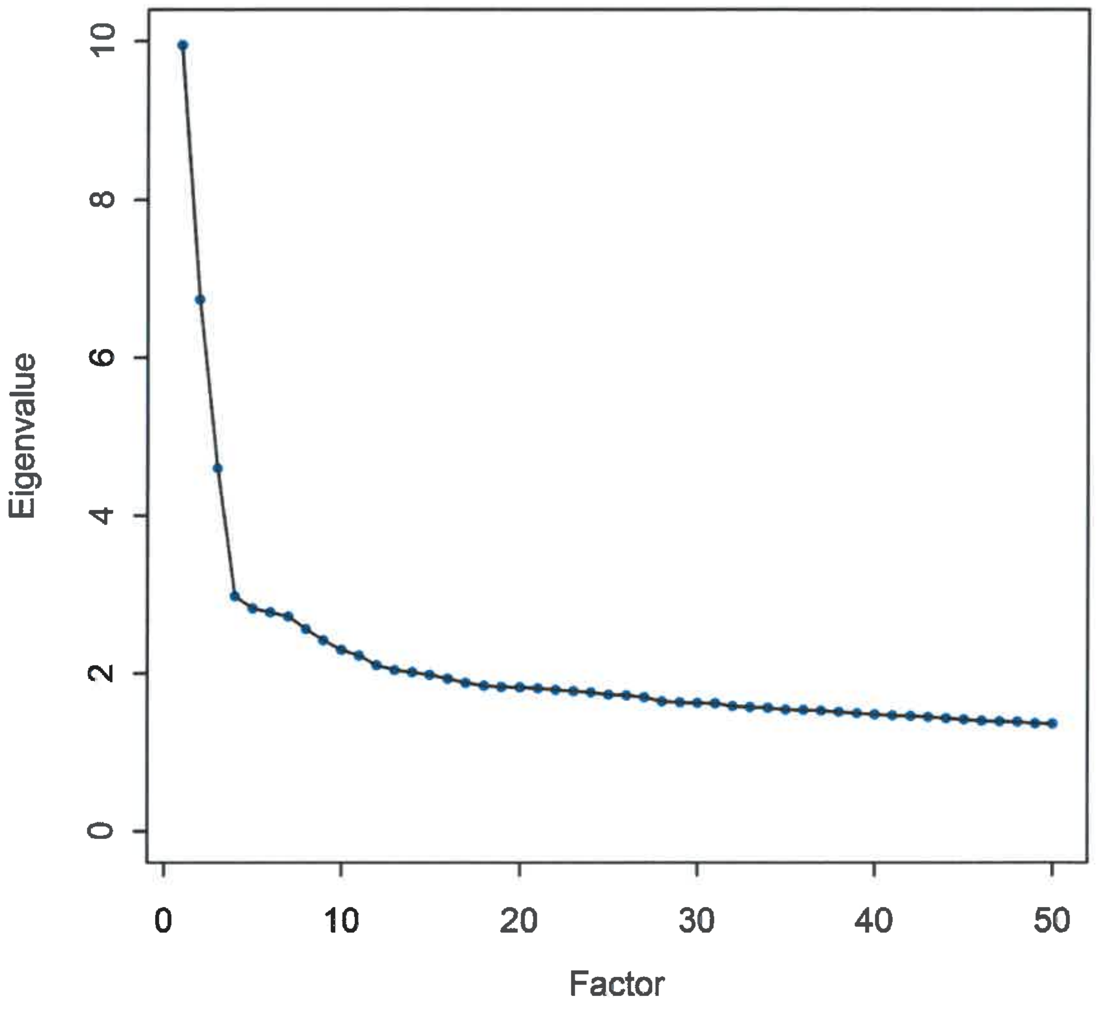

**Supplementary Table 1 - Factor Descriptions.** Provides all ICD-10 codes included in each factor, including their descriptions, frequencies (in TBI and reference population), odds ratio (and 95% confidence interval) from multiple testing results, and factor loading score for each factor.

| Factor Number | Factors | Frequency | | Odds Ratio | Confidence Interval | p-value | Factor Loading |
|---|---|---|---|---|---|---|---|
| | | TBI | Reference Population | | | | |
| 1 | **Cardiology: Cardiovascular Disorders and Other** | **9,818** | **5,434** | **2.2** | **(2.1 , 2.3)** | **<0.0001** | - |
| | I25 Chronic ischemic heart disease | 1,214 | 896 | 1.4 | (1.3 , 1.5) | | 0.72 |
| | I21 ST elevation (STEMI) and non-ST elevation (NSTEMI) myocardial infarction | 505 | 394 | 1.3 | (1.1 , 1.5) | | 0.71 |
| | R94 Abnormal results of function studies | 630 | 369 | 1.7 | (1.5 , 2) | | 0.66 |
| | Z95 Presence of cardiac and vascular implants and grafts | 709 | 357 | 2.0 | (1.8 , 2.3) | | 0.54 |
| | I20 Angina pectoris | 664 | 441 | 1.5 | (1.4 , 1.7) | | 0.51 |
| | E78 Disorders of lipoprotein metabolism and other lipidemias | 566 | 416 | 1.4 | (1.2 , 1.6) | | 0.47 |
| | I24 Other acute ischemic heart diseases | 383 | 189 | 2.0 | (1.7 , 2.4) | | 0.45 |
| | I10 Essential (primary) hypertension | 2,972 | 1,915 | 1.7 | (1.6 , 1.8) | | 0.43 |
| | I50 Heart failure | 1,024 | 473 | 2.3 | (2 , 2.5) | | 0.36 |
| | I48 Atrial fibrillation and flutter | 1,393 | 682 | 2.2 | (2 , 2.5) | | 0.31 |
| | I35 Nonrheumatic aortic valve disorders | 152 | 74 | 2.1 | (1.6 , 2.7) | | 0.24 |
| | R07 Pain in throat and chest | 5,419 | 2,502 | 2.3 | (2.2 , 2.4) | | 0.23 |
| | E11 Type 2 diabetes mellitus | 2,507 | 1,337 | 2.0 | (1.9 , 2.2) | | 0.23 |
| 2 | **Psychiatry: Mental Disorders and Functional Inquiry** | **4,941** | **1,828** | **2.9** | **(2.7 , 3.1)** | **<0.0001** | - |
| | R45 Symptoms and signs involving emotional state | 1,264 | 353 | 3.6 | (3.2 , 4.1) | | 0.50 |
| | F32 Major depressive disorder, single episode | 1,626 | 554 | 3.0 | (2.7 , 3.3) | | 0.48 |
| | F43 Reaction to severe stress, and adjustment disorders | 1,168 | 309 | 3.9 | (3.4 , 4.4) | | 0.48 |
| | F60 Specific personality disorders | 270 | 80 | 3.4 | (2.6 , 4.4) | | 0.45 |
| | Z63 Other problems related to primary support group, including family circumstances | 295 | 105 | 2.8 | (2.2 , 3.5) | | 0.44 |
| | F91 Conduct disorders | 131 | 85 | 1.5 | (1.2 , 2) | | 0.35 |
| | Z91 Personal risk factors, not elsewhere classified | 374 | 108 | 3.5 | (2.8 , 4.3) | | 0.33 |
| | X78 Intentional self-harm by sharp object | 167 | 39 | 4.3 | (3 , 6.1) | | 0.32 |
| | F31 Bipolar disorder | 271 | 90 | 3.0 | (2.4 , 3.8) | | 0.32 |
| | F41 Other anxiety disorders | 1,986 | 739 | 2.8 | (2.6 , 3) | | 0.31 |
| | F33 Major depressive disorder, recurrent | 88 | 42 | 2.1 | (1.5 , 3) | | 0.30 |
| | F34 Persistent mood [affective] disorders | 63 | 37 | 1.7 | (1.1 , 2.6) | | 0.30 |
| | F90 Attention-deficit hyperactivity disorders | 103 | 49 | 2.1 | (1.5 , 3) | | 0.30 |
| | X61 Intentional self-poisoning by and exposure to antiepileptic, sedative-hypnotic, antiparkinsonism and psychotropic drugs, not elsewhere classified | 294 | 72 | 4.1 | (3.2 , 5.3) | | 0.28 |
| | F61 Mixed and other personality disorders | 41 | 7 | 5.9 | (2.6 , 13.1) | | 0.27 |
| | X69 Intentional self-poisoning by and exposure to other and unspecified chemicals and noxious substances | 29 | 8 | 3.6 | (1.7 , 7.9) | | 0.26 |
| | F39 Unspecified mood [affective] disorder | 81 | 21 | 3.9 | (2.4 , 6.2) | | 0.26 |
| | Z59 Problems related to housing and economic circumstances | 158 | 26 | 6.1 | (4 , 9.2) | | 0.25 |
| | F69 Unspecified disorder of adult personality and behaviour | NR | <6 | 10.8 | (4.3 , 27) | | 0.24 |
| | X60 Intentional self-poisoning by and exposure to nonopioid analgesics, antipyretics and antirheumatics | 190 | 48 | 4.0 | (2.9 , 5.4) | | 0.24 |
| | F19 Other psychoactive substance related disorders | 526 | 110 | 4.9 | (4 , 6) | | 0.23 |
| | Z60 Problems related to social environment | 163 | 57 | 2.9 | (2.1 , 3.9) | | 0.23 |
| | T43 Poisoning by, adverse effect of and underdosing of psychotropic drugs, not elsewhere classified | 269 | 61 | 4.4 | (3.3 , 5.8) | | 0.23 |
| | F12 Cannabis related disorders | 157 | 56 | 2.8 | (2.1 , 3.9) | | 0.23 |
| | Z56 Problems related to employment and unemployment | 70 | 12 | 5.8 | (3.2 , 10.8) | | 0.21 |
| | Z65 Problems related to other psychosocial circumstances | 69 | 23 | 3.0 | (1.9 , 4.8) | | 0.20 |
| | T65 Toxic effect of other and unspecified substances | 55 | 22 | 2.5 | (1.5 , 4.1) | | 0.20 |
| 3 | **Geriatry: Disorders of Ederly and Medical Issues** | **14,071** | **6,446** | **2.8** | **(2.7 , 2.9)** | **<0.0001** | - |
| | Z75 Problems related to medical facilities and other health care | 824 | 363 | 2.4 | (2.1 , 2.7) | | 0.43 |
| | R29 Other symptoms and signs involving the nervous and musculoskeletal systems | 459 | 71 | 7.0 | (5.4 , 9) | | 0.41 |
| | S72 Fracture of femur | 429 | 167 | 2.6 | (2.2 , 3.2) | | 0.41 |
| | F03 Unspecified dementia | 422 | 112 | 4.0 | (3.2 , 4.9) | | 0.36 |
| | R64 Cachexia | 212 | 57 | 3.8 | (2.8 , 5.1) | | 0.33 |
| | G20 Parkinson disease | 124 | 31 | 4.0 | (2.7 , 5.9) | | 0.32 |
| | F05 Delirium, not induced by alcohol and other psychoactive substances | 455 | 102 | 4.6 | (3.7 , 5.8) | | 0.32 |

| | | | | | | | | |
|---|---|---|---|---|---|---|---|---|
| | R41 | Other symptoms and signs involving cognitive functions and awareness | 823 | 236 | 3.6 | (3.1 , 4.2) | | 0.30 |
| | W19 | Unspecified fall | 3,376 | 848 | 4.2 | (3.9 , 4.5) | | 0.29 |
| | W05 | Fall from non-moving wheelchair, nonmotorized scooter and motorized mobility scooter | 180 | 30 | 6.0 | (4.1 , 8.8) | | 0.28 |
| | W01 | Fall on same level from slipping, tripping and stumbling | 4,015 | 1,139 | 3.8 | (3.6 , 4.1) | | 0.27 |
| | R26 | Abnormalities of gait and mobility | 228 | 60 | 3.8 | (2.9 , 5) | | 0.26 |
| | W18 | Other slipping, tripping and stumbling and falls | 2,258 | 500 | 4.7 | (4.3 , 5.2) | | 0.26 |
| | F02 | Dementia in other diseases classified elsewhere | NR | <6 | 8.0 | (2.4 , 26.6) | | 0.26 |
| | M81 | Osteoporosis without current pathological fracture | 131 | 79 | 1.7 | (1.3 , 2.2) | | 0.25 |
| | Z50 | Care involving use of rehabilitation procedures | 240 | 105 | 2.3 | (1.8 , 2.9) | | 0.24 |
| | I10 | Essential (primary) hypertension | 2,972 | 1,915 | 1.7 | (1.6 , 1.8) | | 0.23 |
| | N39 | Other disorders of urinary system | 3,480 | 1,965 | 1.9 | (1.8 , 2) | | 0.23 |
| | Z74 | Problems related to care provider dependency | 109 | 30 | 3.6 | (2.4 , 5.4) | | 0.22 |
| | W06 | Fall from bed | 390 | 60 | 6.6 | (5 , 8.7) | | 0.22 |
| | E87 | Other disorders of fluid, electrolyte and acid-base balance | 996 | 421 | 2.5 | (2.2 , 2.8) | | 0.21 |
| | Z73 | Problems related to life management difficulty | 293 | 74 | 4.0 | (3.1 , 5.2) | | 0.21 |
| | R53 | Malaise and fatigue | 1,406 | 627 | 2.3 | (2.1 , 2.6) | | 0.21 |
| 4 | **Trauma: Orthopedic Injuries and Other** | | **22,429** | **8,275** | **4.1** | **(4 , 4.2)** | **<0.0001** | **-** |
| | S62 | Fracture at wrist and hand level | 2,541 | 827 | 3.3 | (3 , 3.6) | | 0.47 |
| | S69 | Other and unspecified injuries of wrist, hand and finger(s) | 1,906 | 771 | 2.5 | (2.3 , 2.8) | | 0.46 |
| | W22 | Striking against or struck by other objects | 5,442 | 1,200 | 5.1 | (4.8 , 5.5) | | 0.41 |
| | S60 | Superficial injury of wrist, hand and fingers | 2,818 | 826 | 3.6 | (3.3 , 3.9) | | 0.41 |
| | S63 | Dislocation and sprain of joints and ligaments at wrist and hand level | 1,805 | 563 | 3.4 | (3 , 3.7) | | 0.40 |
| | S52 | Fracture of forearm | 1,801 | 807 | 2.3 | (2.1 , 2.5) | | 0.39 |
| | W21 | Striking against or struck by sports equipment | 2,084 | 379 | 6.0 | (5.3 , 6.7) | | 0.38 |
| | Z47 | Orthopedic aftercare | 845 | 337 | 2.6 | (2.3 , 2.9) | | 0.36 |
| | W51 | Accidental striking against or bumped into by another person | 2,684 | 553 | 5.5 | (5 , 6.1) | | 0.35 |
| | W02 | Fall involving ice-skates, skis, roller-skates or skateboards | 1,561 | 499 | 3.2 | (2.9 , 3.6) | | 0.35 |
| | W23 | Caught, crushed, jammed or pinched in or between objects | 1,351 | 460 | 3.0 | (2.7 , 3.3) | | 0.32 |
| | S50 | Superficial injury of elbow and forearm | 1,283 | 308 | 4.3 | (3.8 , 4.9) | | 0.27 |
| | W19 | Unspecified fall | 3,376 | 848 | 4.2 | (3.9 , 4.5) | | 0.26 |
| | S59 | Other and unspecified injuries of elbow and forearm | 571 | 221 | 2.6 | (2.2 , 3) | | 0.25 |
| | V18 | Pedal cycle rider injured in noncollision transport accident | 1,045 | 316 | 3.4 | (3 , 3.8) | | 0.25 |
| | X59 | Exposure to unspecified factor | 4,311 | 1,366 | 3.4 | (3.1 , 3.6) | | 0.22 |
| | W18 | Other slipping, tripping and stumbling and falls | 2,258 | 500 | 4.7 | (4.3 , 5.2) | | 0.22 |
| | W01 | Fall on same level from slipping, tripping and stumbling | 4,015 | 1,139 | 3.8 | (3.6 , 4.1) | | 0.22 |
| | S90 | Superficial injury of ankle, foot and toes | 2,093 | 681 | 3.2 | (2.9 , 3.5) | | 0.22 |
| | X50 | Overexertion and strenuous or repetitive movements | 4,765 | 1,870 | 2.7 | (2.6 , 2.9) | | 0.21 |
| | W50 | Accidental hit, strike, kick, twist, bite or scratch by another person | 926 | 158 | 6.0 | (5.1 , 7.1) | | 0.21 |
| 5 | **Nephrology: Disorders of Renal Function and Therapy** | | **1,160** | **535** | **2.3** | **(2.1 , 2.5)** | **<0.0001** | **-** |
| | N18 | Chronic kidney disease (CKD) | 470 | 187 | 2.6 | (2.2 , 3.1) | | 0.70 |
| | Z49 | Encounter for care involving renal dialysis | 75 | 29 | 2.6 | (1.7 , 4) | | 0.68 |
| | Z99 | Dependence on enabling machines and devices, not elsewhere classified | 99 | 33 | 3.0 | (2 , 4.4) | | 0.65 |
| | I12 | Hypertensive chronic kidney disease | 151 | 80 | 1.9 | (1.5 , 2.5) | | 0.60 |
| | N08 | Glomerular disorders in diseases classified elsewhere | 297 | 84 | 3.6 | (2.8 , 4.6) | | 0.56 |
| | N19 | Unspecified kidney failure | 176 | 94 | 1.9 | (1.5 , 2.4) | | 0.36 |
| | T82 | Complications of cardiac and vascular prosthetic devices, implants and grafts | 198 | 110 | 1.8 | (1.4 , 2.3) | | 0.33 |
| | N17 | Acute kidney failure | 637 | 234 | 2.8 | (2.4 , 3.3) | | 0.31 |
| 6 | **Dermatology/Osteopathy/Orthopedy: Skin/Superficial Lesions, Vascular/Lymphatic Pathology, and Back Pain** | | **15,166** | **8,237** | **2.2** | **(2.1 , 2.3)** | **<0.0001** | **-** |
| | L03 | Cellulitis and acute lymphangitis | 2,334 | 999 | 2.4 | (2.2 , 2.6) | | 0.45 |
| | Z51 | Encounter for other aftercare | 1,217 | 608 | 2.0 | (1.8 , 2.2) | | 0.41 |
| | M79 | Other and unspecified soft tissue disorders, not elsewhere classified | 2,935 | 1,386 | 2.2 | (2.1 , 2.3) | | 0.32 |
| | L02 | Cutaneous abscess, furuncle and carbuncle | 607 | 242 | 2.5 | (2.2 , 2.9) | | 0.27 |
| | M25 | Other joint disorder, not elsewhere classified | 1,896 | 895 | 2.1 | (2 , 2.3) | | 0.27 |
| | M54 | Dorsalgia | 3,550 | 1,486 | 2.5 | (2.4 , 2.7) | | 0.26 |
| | R22 | Localized swelling, mass and lump of skin and subcutaneous tissue | 777 | 417 | 1.9 | (1.7 , 2.1) | | 0.25 |
| | L08 | Other local infections of skin and subcutaneous tissue | 504 | 193 | 2.6 | (2.2 , 3.1) | | 0.25 |
| | Z09 | Follow-up examination after treatment for conditions other than malignant neoplasms | 1,653 | 1,187 | 1.4 | (1.3 , 1.5) | | 0.25 |

| | | | | | | | | |
|---|---|---|---|---|---|---|---|---|
| | I80 | Phlebitis and thrombophlebitis | 488 | 241 | 2.1 | (1.8 , 2.4) | | 0.24 |
| | R07 | Pain in throat and chest | 5,419 | 2,502 | 2.3 | (2.2 , 2.4) | | 0.22 |
| | Z48 | Encounter for other postprocedural aftercare | 1,325 | 607 | 2.2 | (2 , 2.5) | | 0.22 |
| | Z76 | Persons encountering health services in other circumstances | 1,082 | 416 | 2.7 | (2.4 , 3) | | 0.21 |
| | L97 | Non-pressure chronic ulcer of lower limb, not elsewhere classified | 127 | 66 | 1.9 | (1.4 , 2.6) | | 0.21 |
| 7 | **Environmental Exposures: Burns** | | **661** | **262** | **2.6** | **(2.2 , 2.9)** | **<0.0001** | - |
| | T31 | Burns classified according to extent of body surface involved | 404 | 148 | 2.8 | (2.3 , 3.3) | | 0.87 |
| | T23 | Burn and corrosion of wrist and hand | 249 | 107 | 2.3 | (1.9 , 2.9) | | 0.62 |
| | X10 | Contact with hot drinks, food, fats and cooking oils | 129 | 53 | 2.5 | (1.8 , 3.4) | | 0.51 |
| | T22 | Burn and corrosion of shoulder and upper limb, except wrist and hand | 108 | 42 | 2.6 | (1.8 , 3.7) | | 0.45 |
| | X12 | Contact with other hot fluids | 78 | 42 | 1.9 | (1.3 , 2.7) | | 0.42 |
| | T21 | Burn and corrosion of trunk | 69 | 21 | 3.4 | (2.1 , 5.6) | | 0.39 |
| | T24 | Burn and corrosion of lower limb, except ankle and foot | 85 | 34 | 2.5 | (1.7 , 3.7) | | 0.38 |
| | X15 | Contact with hot household appliances | 63 | 16 | 3.9 | (2.3 , 6.8) | | 0.37 |
| | T20 | Burn and corrosion of head, face, and neck | 68 | 24 | 2.8 | (1.8 , 4.5) | | 0.34 |
| | T25 | Burn and corrosion of ankle and foot | 60 | 22 | 2.7 | (1.7 , 4.4) | | 0.32 |
| | X09 | Exposure to unspecified smoke, fire and flames | 59 | 18 | 3.3 | (1.9 , 5.6) | | 0.25 |
| | X19 | Contact with other heat and hot substances | 52 | 21 | 2.5 | (1.5 , 4.1) | | 0.23 |
| | X11 | Contact with hot tap-water | 26 | 14 | 1.9 | (1 , 3.6) | | 0.23 |
| | X08 | Exposure to other specified smoke, fire and flames | 60 | 11 | 5.5 | (2.9 , 10.4) | | 0.20 |
| 8 | **Otolaryngology: Respiratory Infections of Upper Airway, Ear and Nose** | | **13,054** | **7,749** | **2.0** | **(2 , 2.1)** | **<0.0001** | - |
| | H66 | Suppurative and unspecified otitis media | 2,396 | 1,474 | 1.8 | (1.6 , 1.9) | | 0.54 |
| | J06 | Acute upper respiratory infections of multiple and unspecified sites | 4,544 | 2,480 | 2.0 | (1.9 , 2.1) | | 0.48 |
| | J02 | Acute pharyngitis | 3,633 | 1,959 | 2.0 | (1.9 , 2.1) | | 0.41 |
| | R50 | Fever of other and unknown origin | 1,763 | 1,282 | 1.4 | (1.3 , 1.5) | | 0.39 |
| | H92 | Otalgia and effusion of ear | 871 | 642 | 1.4 | (1.2 , 1.5) | | 0.38 |
| | B34 | Viral infection of unspecified site | 2,207 | 1,024 | 2.3 | (2.1 , 2.4) | | 0.33 |
| | R21 | Rash and other nonspecific skin eruption | 1,307 | 810 | 1.6 | (1.5 , 1.8) | | 0.31 |
| | R05 | Cough | 1,694 | 1,169 | 1.5 | (1.4 , 1.6) | | 0.31 |
| | J05 | Acute obstructive laryngitis [croup] and epiglottitis | 503 | 283 | 1.8 | (1.6 , 2.2) | | 0.30 |
| | J03 | Acute tonsillitis | 781 | 464 | 1.7 | (1.5 , 1.9) | | 0.29 |
| | H60 | Otitis externa | 889 | 451 | 2.0 | (1.8 , 2.2) | | 0.26 |
| | H10 | Conjunctivitis | 1,057 | 608 | 1.8 | (1.6 , 2) | | 0.25 |
| | J20 | Acute bronchitis | 796 | 408 | 2.0 | (1.8 , 2.2) | | 0.23 |
| 9 | **Gastroenterology: Liver Disorders and Other** | | **417** | **132** | **3.2** | **(2.6 , 3.9)** | **<0.0001** | - |
| | K74 | Fibrosis and cirrhosis of liver | 107 | 23 | 4.7 | (3 , 7.3) | | 0.72 |
| | K72 | Hepatic failure, not elsewhere classified | 64 | 12 | 5.7 | (3 , 10.9) | | 0.61 |
| | R18 | Ascites | 105 | 20 | 5.3 | (3.3 , 8.5) | | 0.60 |
| | I85 | Esophageal varices | 59 | 20 | 3.1 | (1.8 , 5.1) | | 0.60 |
| | K76 | Other diseases of liver | 129 | 43 | 3.0 | (2.1 , 4.2) | | 0.59 |
| | K70 | Alcoholic liver disease | 143 | 23 | 6.5 | (4.1 , 10.1) | | 0.55 |
| | B18 | Chronic viral hepatitis | 86 | 26 | 3.5 | (2.2 , 5.5) | | 0.39 |
| | D61 | Other aplastic anemias and other bone marrow failure syndromes | 55 | 20 | 2.8 | (1.6 , 4.6) | | 0.21 |
| 10 | **Emergency Medicine: Pulmonary, Abdominal and Other** | | **3,237** | **1,766** | **2.0** | **(1.9 , 2.1)** | **<0.0001** | - |
| | A41 | Other sepsis | 299 | 124 | 2.5 | (2 , 3.1) | | 0.52 |
| | J17 | Pneumonia in diseases classified elsewhere | 49 | 27 | 1.8 | (1.1 , 2.9) | | 0.46 |
| | J96 | Respiratory failure, not elsewhere classified | 121 | 70 | 1.7 | (1.3 , 2.3) | | 0.42 |
| | R57 | Shock, not elsewhere classified | 68 | 41 | 1.7 | (1.1 , 2.4) | | 0.40 |
| | N17 | Acute kidney failure | 637 | 234 | 2.8 | (2.4 , 3.3) | | 0.32 |
| | B95 | Streptococcus, Staphylococcus, and Enterococcus as the cause of diseases classified elsewhere | 449 | 267 | 1.7 | (1.5 , 2) | | 0.30 |
| | B96 | Other bacterial agents as the cause of diseases classified elsewhere | 898 | 624 | 1.5 | (1.3 , 1.6) | | 0.27 |
| | E87 | Other disorders of fluid, electrolyte and acid-base balance | 996 | 421 | 2.5 | (2.2 , 2.8) | | 0.27 |
| | L89 | Pressure ulcer | 61 | 28 | 2.2 | (1.4 , 3.4) | | 0.26 |
| | F05 | Delirium, not induced by alcohol and other psychoactive substances | 455 | 102 | 4.6 | (3.7 , 5.8) | | 0.25 |
| | U82 | Resistance to betalactam antibiotics | 79 | 29 | 2.7 | (1.8 , 4.2) | | 0.25 |
| | J15 | Bacterial pneumonia, not elsewhere classified | 64 | 26 | 2.5 | (1.6 , 3.9) | | 0.23 |
| | Z75 | Problems related to medical facilities and other health care | 824 | 363 | 2.4 | (2.1 , 2.7) | | 0.23 |
| | J90 | Pleural effusion, not elsewhere classified | 237 | 115 | 2.1 | (1.7 , 2.6) | | 0.22 |

| | | | | | | | | |
|---|---|---|---|---|---|---|---|---|
| | J69 | Pneumonitis due to solids and liquids | 100 | 32 | 3.1 | (2.1 , 4.7) | | 0.20 |
| | A49 | Bacterial infection of unspecified site | 96 | 39 | 2.5 | (1.7 , 3.6) | | 0.20 |
| 11 | | **Gastroenterology: Metabolic Disorders and Adbominal Symptoms** | **13,138** | **8,072** | **1.9** | **(1.8 , 2)** | **<0.0001** | - |
| | K29 | Gastritis and duodenitis | 1,264 | 892 | 1.4 | (1.3 , 1.6) | | 0.38 |
| | D63 | Anemia in chronic diseases classified elsewhere | 123 | 44 | 2.8 | (2 , 3.9) | | 0.34 |
| | D64 | Other anemias | 889 | 510 | 1.8 | (1.6 , 2) | | 0.33 |
| | K92 | Other diseases of digestive system | 847 | 415 | 2.1 | (1.8 , 2.3) | | 0.31 |
| | Z85 | Personal history of malignant neoplasm | 812 | 721 | 1.1 | (1 , 1.3) | | 0.27 |
| | E87 | Other disorders of fluid, electrolyte and acid-base balance | 996 | 421 | 2.5 | (2.2 , 2.8) | | 0.26 |
| | D50 | Iron deficiency anemia | 255 | 181 | 1.4 | (1.2 , 1.7) | | 0.26 |
| | D46 | Myelodysplastic syndromes | NR | <6 | 8.8 | (3.1 , 24.6) | | 0.25 |
| | E86 | Volume depletion | 898 | 401 | 2.3 | (2 , 2.6) | | 0.25 |
| | R11 | Nausea and vomiting | 2,135 | 1,132 | 1.9 | (1.8 , 2.1) | | 0.25 |
| | E83 | Disorders of mineral metabolism | 168 | 72 | 2.3 | (1.8 , 3.1) | | 0.23 |
| | K21 | Gastro-esophageal reflux disease | 926 | 640 | 1.5 | (1.3 , 1.6) | | 0.23 |
| | I10 | Essential (primary) hypertension | 2,972 | 1,915 | 1.7 | (1.6 , 1.8) | | 0.21 |
| | D61 | Other aplastic anemias and other bone marrow failure syndromes | 55 | 20 | 2.8 | (1.6 , 4.6) | | 0.21 |
| | R10 | Abdominal and pelvic pain | 6,999 | 3,715 | 2.1 | (2 , 2.1) | | 0.21 |
| | K56 | Paralytic ileus and intestinal obstruction without hernia | 353 | 251 | 1.4 | (1.2 , 1.7) | | 0.21 |
| | N17 | Acute kidney failure | 637 | 234 | 2.8 | (2.4 , 3.3) | | 0.21 |
| | D69 | Purpura and other hemorrhagic conditions | 181 | 72 | 2.5 | (1.9 , 3.3) | | 0.20 |
| | C79 | Secondary malignant neoplasm of other and unspecified sites | 88 | 25 | 3.5 | (2.3 , 5.5) | | 0.20 |
| 12 | | **Neurology: Stroke and Emergencies Involving the Brain** | **3,923** | **2,332** | **1.9** | **(1.8 , 2)** | **<0.0001** | - |
| | G81 | Hemiplegia and hemiparesis | 109 | 49 | 2.2 | (1.6 , 3.1) | | 0.60 |
| | I63 | Cerebral infarction | 302 | 113 | 2.8 | (2.2 , 3.4) | | 0.60 |
| | R47 | Speech disturbances, not elsewhere classified | 157 | 61 | 2.6 | (1.9 , 3.5) | | 0.53 |
| | I64 | Stroke, not specified as haemorrhage or infarction | 435 | 170 | 2.6 | (2.2 , 3.1) | | 0.52 |
| | I69 | Sequelae of cerebrovascular disease | 122 | 40 | 3.1 | (2.2 , 4.5) | | 0.48 |
| | Z75 | Problems related to medical facilities and other health care | 824 | 363 | 2.4 | (2.1 , 2.7) | | 0.27 |
| | I61 | Nontraumatic intracerebral hemorrhage | 73 | 23 | 3.2 | (2 , 5.1) | | 0.27 |
| | G45 | Transient cerebral ischemic attacks and related syndromes | 514 | 224 | 2.4 | (2 , 2.8) | | 0.27 |
| | I67 | Other cerebrovascular diseases | 75 | 26 | 3.0 | (1.9 , 4.7) | | 0.24 |
| | I10 | Essential (primary) hypertension | 2,972 | 1,915 | 1.7 | (1.6 , 1.8) | | 0.24 |
| | I62 | Other and unspecified nontraumatic intracranial hemorrhage | 109 | 23 | 4.7 | (3 , 7.4) | | 0.21 |
| 13 | | **Pharmacology Emergencies: Adverse Drug Effests of Medications Crossing Blood-Brain Barrier** | **508** | **112** | **4.6** | **(3.7 , 5.6)** | **<0.0001** | - |
| | T42 | Poisoning by, adverse effect of and underdosing of antiepileptic, sedative- hypnotic and antiparkinsonism drugs | 295 | 52 | 5.7 | (4.2 , 7.6) | | 0.72 |
| | X61 | Intentional self-poisoning by and exposure to antiepileptic, sedative-hypnotic, antiparkinsonism and psychotropic drugs, not elsewhere classified | 294 | 72 | 4.1 | (3.2 , 5.3) | | 0.69 |
| | T43 | Poisoning by, adverse effect of and underdosing of psychotropic drugs, not elsewhere classified | 269 | 61 | 4.4 | (3.3 , 5.8) | | 0.68 |
| | X41 | Accidental poisoning by and exposure to antiepileptic, sedative-hypnotic, antiparkinsonism and psychotropic drugs, not elsewhere classified | 146 | 32 | 4.6 | (3.1 , 6.7) | | 0.51 |
| | Y11 | Poisoning by and exposure to antiepileptic, sedative-hypnotic, antiparkinsonism and psychotropic drugs, not elsewhere classified, undetermined intent | 106 | 11 | 9.6 | (5.2 , 17.9) | | 0.46 |
| 14 | | **Toxicology: Emergencies and Adversities Due to Substance Abuse** | **4,503** | **1,198** | **4.1** | **(3.9 , 4.4)** | **<0.0001** | - |
| | F19 | Other psychoactive substance related disorders | 526 | 110 | 4.9 | (4 , 6) | | 0.46 |
| | F11 | Opioid related disorders | 244 | 46 | 5.3 | (3.9 , 7.3) | | 0.42 |
| | F14 | Cocaine related disorders | 245 | 43 | 5.7 | (4.1 , 7.9) | | 0.38 |
| | F10 | Alcohol related disorders | 1,849 | 374 | 5.2 | (4.6 , 5.8) | | 0.34 |
| | Z76 | Persons encountering health services in other circumstances | 1,082 | 416 | 2.7 | (2.4 , 3) | | 0.30 |
| | Y04 | Assault by bodily force | 1,564 | 140 | 12.0 | (10.1 , 14.4) | | 0.30 |
| | Z72 | Problems related to lifestyle | 544 | 186 | 3.0 | (2.5 , 3.5) | | 0.30 |
| | Z59 | Problems related to housing and economic circumstances | 158 | 26 | 6.1 | (4 , 9.2) | | 0.27 |
| | F13 | Sedative, hypnotic, or anxiolytic related disorders | 81 | 13 | 6.2 | (3.5 , 11.2) | | 0.25 |
| | Y09 | Assault by unspecified means | 130 | 7 | 18.6 | (8.7 , 39.7) | | 0.20 |
| 15 | | **Endocrinology: Diabetes and Diabetic Consequences** | **3,336** | **1,938** | **1.9** | **(1.8 , 2)** | **<0.0001** | - |
| | E14 | Unspecified diabetes mellitus | 1,720 | 920 | 1.9 | (1.8 , 2.1) | | 0.63 |
| | E11 | Type 2 diabetes mellitus | 2,507 | 1,337 | 2.0 | (1.9 , 2.2) | | 0.59 |

| | | | | | | | | |
|---|---|---|---|---|---|---|---|---|
| | E10 | Type 1 diabetes mellitus | 429 | 213 | 2.0 | (1.7 , 2.4) | | 0.55 |
| | R73 | Elevated blood glucose level | 414 | 254 | 1.6 | (1.4 , 1.9) | | 0.54 |
| | H36 | Retinal disorders in diseases classified elsewhere | 67 | 36 | 1.9 | (1.2 , 2.8) | | 0.34 |
| | N08 | Glomerular disorders in diseases classified elsewhere | 297 | 84 | 3.6 | (2.8 , 4.6) | | 0.33 |
| | I79 | Disorders of arteries, arterioles and capillaries in diseases classified elsewhere | 59 | 23 | 2.6 | (1.6 , 4.2) | | 0.33 |
| | L97 | Non-pressure chronic ulcer of lower limb, not elsewhere classified | 127 | 66 | 1.9 | (1.4 , 2.6) | | 0.31 |
| | G63 | Polyneuropathy in diseases classified elsewhere | 72 | 21 | 3.4 | (2.1 , 5.6) | | 0.30 |
| | M86 | Osteomyelitis | 93 | 44 | 2.1 | (1.5 , 3) | | 0.26 |
| 16 | | **Gastroenterology and Obstetrics: Conditions and Symptoms of Abdomen and Pelvis** | **12,554** | **7,130** | **2.0** | **(2 , 2.1)** | **<0.0001** | - |
| | R10 | Abdominal and pelvic pain | 6,999 | 3,715 | 2.1 | (2 , 2.1) | | 0.48 |
| | N83 | Noninflammatory disorders of ovary, fallopian tube and broad ligament | 453 | 192 | 2.4 | (2 , 2.8) | | 0.40 |
| | Z33 | Pregnant state | 321 | 225 | 1.5 | (1.2 , 1.7) | | 0.36 |
| | N93 | Other abnormal uterine and vaginal bleeding | 403 | 274 | 1.5 | (1.3 , 1.8) | | 0.32 |
| | Z32 | Encounter for pregnancy test and childbirth and childcare instruction | 150 | 87 | 1.7 | (1.3 , 2.3) | | 0.30 |
| | N94 | Pain and other conditions associated with female genital organs and menstrual cycle | 244 | 141 | 1.7 | (1.4 , 2.1) | | 0.30 |
| | N73 | Other female pelvic inflammatory diseases | 122 | 70 | 1.7 | (1.3 , 2.3) | | 0.30 |
| | N39 | Other disorders of urinary system | 3,480 | 1,965 | 1.9 | (1.8 , 2) | | 0.27 |
| | K37 | Unspecified appendicitis | 370 | 117 | 3.2 | (2.6 , 3.9) | | 0.26 |
| | Z71 | Persons encountering health services for other counseling and medical advice, not elsewhere classified | 1,813 | 757 | 2.5 | (2.3 , 2.7) | | 0.25 |
| | G43 | Migraine | 1,028 | 315 | 3.3 | (2.9 , 3.8) | | 0.23 |
| | K35 | Acute appendicitis | 315 | 258 | 1.2 | (1 , 1.4) | | 0.22 |
| | N12 | Tubulo-interstitial nephritis, not specified as acute or chronic | 424 | 179 | 2.4 | (2 , 2.8) | | 0.22 |
| | R11 | Nausea and vomiting | 2,135 | 1,132 | 1.9 | (1.8 , 2.1) | | 0.21 |
| 17 | | **Cardiology: Chronic Cardiovascular Pathology and Other** | **2,615** | **1,339** | **2.2** | **(2 , 2.4)** | **<0.0001** | - |
| | Y44 | Agents primarily affecting blood constituents | 159 | 66 | 2.4 | (1.8 , 3.2) | | 0.63 |
| | D68 | Other coagulation defects | 194 | 70 | 2.8 | (2.1 , 3.6) | | 0.55 |
| | R78 | Findings of drugs and other substances, not normally found in blood | 166 | 54 | 3.2 | (2.3 , 4.4) | | 0.53 |
| | Z92 | Personal history of medical treatment | 447 | 185 | 2.5 | (2.1 , 2.9) | | 0.47 |
| | I48 | Atrial fibrillation and flutter | 1,393 | 682 | 2.2 | (2 , 2.5) | | 0.42 |
| | I50 | Heart failure | 1,024 | 473 | 2.3 | (2 , 2.5) | | 0.33 |
| | I80 | Phlebitis and thrombophlebitis | 488 | 241 | 2.1 | (1.8 , 2.4) | | 0.21 |
| 18 | | **Trauma: Superficial Injuries and Other** | **8,638** | **3,067** | **3.2** | **(3.1 , 3.3)** | **<0.0001** | - |
| | V43 | Car occupant injured in collision with car, pick-up truck or van | 899 | 231 | 4.0 | (3.5 , 4.6) | | 0.37 |
| | S20 | Superficial injury of thorax | 1,209 | 296 | 4.2 | (3.7 , 4.8) | | 0.28 |
| | S30 | Superficial injury of abdomen, lower back, pelvis and external genitals | 868 | 221 | 4.0 | (3.4 , 4.6) | | 0.28 |
| | S19 | Other specified and unspecified injuries of neck | 329 | 81 | 4.1 | (3.2 , 5.3) | | 0.26 |
| | S10 | Superficial injury of neck | 368 | 97 | 3.8 | (3.1 , 4.8) | | 0.26 |
| | S39 | Other and unspecified injuries of abdomen, lower back, pelvis and external genitals | 799 | 227 | 3.6 | (3.1 , 4.2) | | 0.26 |
| | T00 | Superficial injuries involving multiple body regions | 551 | 155 | 3.6 | (3 , 4.3) | | 0.26 |
| | M54 | Dorsalgia | 3,550 | 1,486 | 2.5 | (2.4 , 2.7) | | 0.25 |
| | S40 | Superficial injury of shoulder and upper arm | 1,034 | 250 | 4.2 | (3.7 , 4.8) | | 0.25 |
| | V89 | Motor- or nonmotor-vehicle accident, type of vehicle unspecified | 135 | 32 | 4.2 | (2.9 , 6.2) | | 0.23 |
| | T14 | Injury of unspecified body region | 428 | 161 | 2.7 | (2.2 , 3.2) | | 0.22 |
| | S49 | Other and unspecified injuries of shoulder and upper arm | 773 | 237 | 3.3 | (2.8 , 3.8) | | 0.22 |
| | T09 | Other injuries of spine and trunk, level unspecified | 411 | 106 | 3.9 | (3.2 , 4.9) | | 0.20 |
| 19 | | **Infectious Diseases and Respirology: Acute and Chronic Disorders of Airway and Lung** | **13,938** | **7,653** | **2.1** | **(2.1 , 2.2)** | **<0.0001** | - |
| | R06 | Abnormalities of breathing | 1,933 | 1,076 | 1.8 | (1.7 , 2) | | 0.45 |
| | J45 | Asthma | 1,811 | 1,029 | 1.8 | (1.7 , 1.9) | | 0.42 |
| | J18 | Pneumonia, unspecified organism | 2,553 | 1,203 | 2.2 | (2.1 , 2.4) | | 0.42 |
| | R05 | Cough | 1,694 | 1,169 | 1.5 | (1.4 , 1.6) | | 0.37 |
| | J44 | Other chronic obstructive pulmonary disease | 849 | 467 | 1.9 | (1.7 , 2.1) | | 0.35 |
| | J98 | Other respiratory disorders | 693 | 331 | 2.1 | (1.9 , 2.4) | | 0.31 |
| | J06 | Acute upper respiratory infections of multiple and unspecified sites | 4,544 | 2,480 | 2.0 | (1.9 , 2.1) | | 0.29 |

| | | | | | | | | |
|---|---|---|---|---|---|---|---|---|
| | J40 | Bronchitis, not specified as acute or chronic | 1,227 | 592 | 2.1 | (1.9 , 2.3) | | 0.28 |
| | J20 | Acute bronchitis | 796 | 408 | 2.0 | (1.8 , 2.2) | | 0.23 |
| | R07 | Pain in throat and chest | 5,419 | 2,502 | 2.3 | (2.2 , 2.4) | | 0.20 |
| 20 | **Toxicology: Alcohol-Related Emergencies** | | **186** | **36** | **5.2** | **(3.6 , 7.4)** | **<0.0001** | - |
| | T51 | Toxic effect of alcohol | 135 | 27 | 5.0 | (3.3 , 7.6) | | 0.90 |
| | X45 | Accidental poisoning by and exposure to alcohol | 56 | 13 | 4.3 | (2.4 , 7.9) | | 0.66 |
| | Y15 | Poisoning by and exposure to alcohol, undetermined intent | NR | <6 | 10.3 | (3.2 , 33.8) | | 0.56 |
| | X65 | Intentional self-poisoning by and exposure to alcohol | 52 | 11 | 4.7 | (2.5 , 9.1) | | 0.54 |
| | Y90 | Evidence of alcohol involvement determined by blood alcohol level | 54 | 6 | 9.0 | (3.9 , 20.9) | | 0.20 |
| 21 | **Psychiatry: Schizophrenia and Delusional Disorders** | | **1,004** | **334** | **3.1** | **(2.7 , 3.5)** | **<0.0001** | - |
| | F20 | Schizophrenia | 172 | 74 | 2.3 | (1.8 , 3.1) | | 0.65 |
| | F29 | Unspecified nonorganic psychosis | 209 | 88 | 2.4 | (1.9 , 3) | | 0.58 |
| | F25 | Schizoaffective disorders | 51 | 19 | 2.7 | (1.6 , 4.5) | | 0.56 |
| | F22 | Persistent delusional disorders | 109 | 37 | 3.0 | (2.1 , 4.4) | | 0.50 |
| | R44 | Other symptoms and signs involving general sensations and perceptions | 152 | 53 | 2.9 | (2.1 , 3.9) | | 0.38 |
| | R46 | Symptoms and signs involving appearance and behavior | 271 | 67 | 4.0 | (3.1 , 5.3) | | 0.33 |
| | F31 | Bipolar disorder | 271 | 90 | 3.0 | (2.4 , 3.8) | | 0.27 |
| | F99 | Mental disorder, not otherwise specified | 56 | 12 | 4.7 | (2.5 , 8.7) | | 0.25 |
| | Z59 | Problems related to housing and economic circumstances | 158 | 26 | 6.1 | (4 , 9.2) | | 0.21 |
| 22 | **Pharmacology Emergencies: Poisoning due to Narcotics** | | **468** | **82** | **5.7** | **(4.5 , 7.2)** | **<0.0001** | - |
| | T40 | Poisoning by, adverse effect of and underdosing of narcotics and psychodysleptics [hallucinogens] | 249 | 35 | 7.1 | (5 , 10.1) | | 0.85 |
| | X42 | Accidental poisoning by and exposure to narcotics and psychodysleptics [hallucinogens], not elsewhere classified | 121 | 20 | 6.1 | (3.8 , 9.7) | | 0.63 |
| | Y12 | Poisoning by and exposure to narcotics and psychodysleptics [hallucinogens], not elsewhere classified, undetermined intent | 71 | 9 | 7.9 | (3.9 , 15.8) | | 0.54 |
| | X62 | Intentional self-poisoning by and exposure to narcotics and psychodysleptics [hallucinogens], not elsewhere classified | 95 | 13 | 7.3 | (4.1 , 13) | | 0.52 |
| | F11 | Opioid related disorders | 244 | 46 | 5.3 | (3.9 , 7.3) | | 0.23 |
| 23 | **Trauma: Injuries from Contact With Sharp Instruments and Machinery** | | **7,528** | **3,301** | **2.5** | **(2.4 , 2.7)** | **<0.0001** | - |
| | S61 | Open wound of wrist, hand and fingers | 3,463 | 1,658 | 2.2 | (2.1 , 2.3) | | 0.77 |
| | W26 | Contact with knife, sword or dagger | 972 | 441 | 2.2 | (2 , 2.5) | | 0.47 |
| | W45 | Foreign body or object entering through skin | 1,272 | 565 | 2.3 | (2.1 , 2.6) | | 0.45 |
| | W25 | Contact with sharp glass | 885 | 309 | 2.9 | (2.5 , 3.3) | | 0.39 |
| | S91 | Open wound of ankle, foot and toes | 900 | 381 | 2.4 | (2.1 , 2.7) | | 0.34 |
| | W29 | Contact with other powered hand tools and household machinery | 283 | 138 | 2.1 | (1.7 , 2.5) | | 0.28 |
| | W49 | Exposure to other inanimate mechanical forces | 692 | 250 | 2.8 | (2.4 , 3.2) | | 0.28 |
| | W27 | Contact with nonpowered hand tool | 379 | 174 | 2.2 | (1.8 , 2.6) | | 0.26 |
| | S51 | Open wound of elbow and forearm | 669 | 183 | 3.7 | (3.1 , 4.3) | | 0.23 |
| | Z48 | Encounter for other postprocedural aftercare | 1,325 | 607 | 2.2 | (2 , 2.5) | | 0.22 |
| | W31 | Contact with other and unspecified machinery | 257 | 139 | 1.9 | (1.5 , 2.3) | | 0.22 |
| | W23 | Caught, crushed, jammed or pinched in or between objects | 1,351 | 460 | 3.0 | (2.7 , 3.3) | | 0.21 |
| 24 | **Pharmacology Emergencies and Toxicology: Poisoning due to Hormones, Cardiovascular Drugs and Other** | | **420** | **159** | **2.7** | **(2.2 , 3.2)** | **<0.0001** | - |
| | X44 | Accidental poisoning by and exposure to other and unspecified drugs, medicaments and biological substances | 181 | 74 | 2.4 | (1.9 , 3.2) | | 0.60 |
| | T50 | Poisoning by, adverse effect of and underdosing of diuretics and other and unspecified drugs, medicaments and biological substances | 187 | 51 | 3.7 | (2.7 , 5) | | 0.59 |
| | X64 | Intentional self-poisoning by and exposure to other and unspecified drugs, medicaments and biological substances | 132 | 42 | 3.1 | (2.2 , 4.4) | | 0.55 |
| | Y14 | Poisoning by and exposure to other and unspecified drugs, medicaments and biological substances, undetermined intent | 59 | 21 | 2.8 | (1.7 , 4.6) | | 0.44 |
| | T45 | Poisoning by, adverse effect of and underdosing of primarily systemic and hematological agents, not elsewhere classified | 83 | 32 | 2.6 | (1.7 , 3.9) | | 0.42 |
| | T38 | Poisoning by, adverse effect of and underdosing of hormones and their synthetic substitutes and antagonists, not elsewhere classified | 26 | 10 | 2.6 | (1.3 , 5.4) | | 0.40 |
| | T46 | Poisoning by, adverse effect of and underdosing of agents primarily affecting the cardiovascular system | 35 | 18 | 1.9 | (1.1 , 3.4) | | 0.36 |
| | T49 | Poisoning by, adverse effect of and underdosing of topical agents primarily affecting skin and mucous membrane and by ophthalmological, otorhinorlaryngological and dental drugs | 19 | 6 | 3.2 | (1.3 , 7.9) | | 0.29 |

| | | | | | | | | |
|---|---|---|---|---|---|---|---|---|
| 25 | | **Pharmacology Emergencies: Poisoning by Pain Killers and Anti-Inflammatory Drugs** | **295** | **77** | **3.8** | **(3 , 4.9)** | **<0.0001** | **-** |
| | T39 | Poisoning by, adverse effect of and underdosing of nonopioid analgesics, antipyretics and antirheumatics | 274 | 73 | 3.8 | (2.9 , 4.9) | | 0.90 |
| | X60 | Intentional self-poisoning by and exposure to nonopioid analgesics, antipyretics and antirheumatics | 190 | 48 | 4.0 | (2.9 , 5.4) | | 0.70 |
| | X40 | Accidental poisoning by and exposure to nonopioid analgesics, antipyretics and antirheumatics | 76 | 23 | 3.3 | (2.1 , 5.3) | | 0.55 |
| | Y10 | Poisoning by and exposure to nonopioid analgesics, antipyretics and antirheumatics, undetermined intent | 36 | 11 | 3.3 | (1.7 , 6.4) | | 0.39 |
| 26 | | **Neurology: Epilepsy, Seizures, Brain Lesions and Other** | **1,121** | **365** | **3.1** | **(2.8 , 3.5)** | **<0.0001** | **-** |
| | G40 | Epilepsy and recurrent seizures | 390 | 103 | 3.8 | (3 , 4.7) | | 0.64 |
| | R56 | Convulsions, not elsewhere classified | 824 | 264 | 3.2 | (2.7 , 3.6) | | 0.60 |
| | G41 | Status epilepticus | NR | <6 | 6.2 | (2.4 , 15.9) | | 0.46 |
| | Y46 | Antiepileptics and antiparkinsonism drugs | 62 | 8 | 7.8 | (3.7 , 16.2) | | 0.38 |
| | F44 | Dissociative and conversion disorders | 74 | 19 | 3.9 | (2.4 , 6.4) | | 0.33 |
| | D43 | Neoplasm of uncertain behavior of brain and central nervous system | 30 | 9 | 3.3 | (1.6 , 7) | | 0.28 |
| | G93 | Other disorders of brain | 102 | 28 | 3.6 | (2.4 , 5.5) | | 0.26 |
| | C71 | Malignant neoplasm of brain | 37 | 17 | 2.2 | (1.2 , 3.9) | | 0.25 |
| 27 | | **Trauma: Overexertion and Injuries to the Lower Limb** | **12,053** | **4,790** | **3.0** | **(2.9 , 3.1)** | **<0.0001** | **-** |
| | X50 | Overexertion and strenuous or repetitive movements | 4,765 | 1,870 | 2.7 | (2.6 , 2.9) | | 0.53 |
| | S93 | Dislocation and sprain of joints and ligaments at ankle, foot and toe level | 2,931 | 1,123 | 2.8 | (2.6 , 3) | | 0.46 |
| | S99 | Other and unspecified injuries of ankle and foot | 1,357 | 672 | 2.0 | (1.9 , 2.2) | | 0.33 |
| | X59 | Exposure to unspecified factor | 4,311 | 1,366 | 3.4 | (3.1 , 3.6) | | 0.32 |
| | S83 | Dislocation and sprain of joints and ligaments of knee | 1,204 | 458 | 2.7 | (2.4 , 3) | | 0.26 |
| | S90 | Superficial injury of ankle, foot and toes | 2,093 | 681 | 3.2 | (2.9 , 3.5) | | 0.25 |
| | S82 | Fracture of lower leg, including ankle | 1,167 | 482 | 2.5 | (2.2 , 2.7) | | 0.24 |
| | T78 | Adverse effects, not elsewhere classified | 936 | 395 | 2.4 | (2.1 , 2.7) | | 0.24 |
| | X58 | Exposure to other specified factors | 1,127 | 338 | 3.4 | (3 , 3.8) | | 0.23 |
| 28 | | **Nephrology: Genitourinary Disorders, Prosthetic and Other** | **5,379** | **3,436** | **1.7** | **(1.6 , 1.7)** | **<0.0001** | **-** |
| | T83 | Complications of genitourinary prosthetic devices, implants and grafts | 108 | 71 | 1.5 | (1.1 , 2.1) | | 0.56 |
| | R33 | Retention of urine | 469 | 318 | 1.5 | (1.3 , 1.7) | | 0.51 |
| | Z46 | Encounter for fitting and adjustment of other devices | 238 | 196 | 1.2 | (1 , 1.5) | | 0.49 |
| | R31 | Hematuria | 615 | 520 | 1.2 | (1.1 , 1.3) | | 0.41 |
| | Y84 | Other medical procedures as the cause of abnormal reaction of the patient, or of later complication, without mention of misadventure at the time of the procedure | 600 | 296 | 2.1 | (1.8 , 2.4) | | 0.39 |
| | N20 | Calculus of kidney and ureter | 307 | 259 | 1.2 | (1 , 1.4) | | 0.34 |
| | N39 | Other disorders of urinary system | 3,480 | 1,965 | 1.9 | (1.8 , 2) | | 0.34 |
| | N23 | Unspecified renal colic | 487 | 311 | 1.6 | (1.4 , 1.8) | | 0.28 |
| | N30 | Cystitis | 337 | 274 | 1.2 | (1 , 1.4) | | 0.25 |
| | B96 | Other bacterial agents as the cause of diseases classified elsewhere | 898 | 624 | 1.5 | (1.3 , 1.6) | | 0.22 |
| 29 | | **Neurology: Alzheimer's Diseases and Dementia** | **530** | **148** | **3.8** | **(3.2 , 4.6)** | **<0.0001** | **-** |
| | G30 | Alzheimer's disease | 162 | 52 | 3.2 | (2.3 , 4.4) | | 0.92 |
| | F00 | Dementia in Alzheimer disease | 112 | 39 | 2.9 | (2 , 4.2) | | 0.92 |
| | F03 | Unspecified dementia | 422 | 112 | 4.0 | (3.2 , 4.9) | | 0.27 |
| 30 | | **Emergency Medicine: Foreign Body in Eye, Airway and Other** | **1,682** | **624** | **2.8** | **(2.5 , 3.1)** | **<0.0001** | **-** |
| | W44 | Foreign body entering into or through eye or natural orifice | 1,564 | 560 | 2.9 | (2.6 , 3.2) | | 0.91 |
| | T15 | Foreign body on external eye | 627 | 300 | 2.2 | (1.9 , 2.5) | | 0.63 |
| | T18 | Foreign body in alimentary tract | 239 | 93 | 2.6 | (2 , 3.3) | | 0.45 |
| | T17 | Foreign body in respiratory tract | 187 | 111 | 1.7 | (1.3 , 2.1) | | 0.42 |
| | T16 | Foreign body in ear | 144 | 58 | 2.5 | (1.8 , 3.4) | | 0.30 |
| | H18 | Other disorders of cornea | 82 | 42 | 2.0 | (1.4 , 2.9) | | 0.22 |
| 31 | | **Emergency Medicine: Complications of Medical Procedures** | **2,600** | **1,533** | **1.8** | **(1.6 , 1.9)** | **<0.0001** | **-** |
| | Y83 | Surgical operation and other surgical procedures as the cause of abnormal reaction of the patient, or of later complication, without mention of misadventure at the time of the procedure | 2,149 | 1,276 | 1.7 | (1.6 , 1.9) | | 0.70 |
| | T81 | Complications of procedures, not elsewhere classified | 1,315 | 763 | 1.8 | (1.6 , 1.9) | | 0.68 |
| | Y84 | Other medical procedures as the cause of abnormal reaction of the patient, or of later complication, without mention of misadventure at the time of the procedure | 600 | 296 | 2.1 | (1.8 , 2.4) | | 0.34 |
| | T82 | Complications of cardiac and vascular prosthetic devices, implants and grafts | 198 | 110 | 1.8 | (1.4 , 2.3) | | 0.32 |

| | | | | | | | | |
|---|---|---|---|---|---|---|---|---|
| 32 | | **Environmental Exposures: Exposure to Heat and Light** | **66** | **26** | **2.5** | **(1.6 , 4)** | **<0.0001** | - |
| | X30 | Exposure to excessive natural heat | 54 | 18 | 3.0 | (1.8 , 5.1) | | 0.95 |
| | T67 | Effects of heat and light | 62 | 25 | 2.5 | (1.6 , 3.9) | | 0.95 |
| 33 | | **Environmental Exposures: Exposure to Cold/Hypothermia** | **82** | **21** | **3.9** | **(2.4 , 6.3)** | **<0.0001** | - |
| | X31 | Exposure to excessive natural cold | 76 | 19 | 4.0 | (2.4 , 6.6) | | 0.93 |
| | T35 | Frostbite involving multiple body regions and unspecified frostbite | 39 | 11 | 3.5 | (1.8 , 6.9) | | 0.77 |
| | T68 | Hypothermia | NR | <6 | 4.0 | (1.5 , 10.7) | | 0.53 |
| 34 | | **Environmental Exposures: Bee, Wasp and Hornet Stings** | **349** | **174** | **2.0** | **(1.7 , 2.4)** | **<0.0001** | - |
| | T63 | Toxic effect of contact with venomous animals and plants | 267 | 139 | 1.9 | (1.6 , 2.4) | | 0.94 |
| | X23 | Contact with hornets, wasps and bees | 314 | 156 | 2.0 | (1.7 , 2.4) | | 0.94 |
| 35 | | **Infectious Diseases: Viral Conjunctivitis** | **84** | **41** | **2.0** | **(1.4 , 3)** | **0.0002** | - |
| | B30 | Viral conjunctivitis | 80 | 39 | 2.1 | (1.4 , 3) | | 0.94 |
| | H13 | Disorders of conjunctiva in diseases classified elsewhere | 54 | 24 | 2.3 | (1.4 , 3.6) | | 0.94 |
| 36 | | **Trauma: Assault and Intentional Injury** | **1,266** | **334** | **3.9** | **(3.4 , 4.4)** | **<0.0001** | - |
| | X99 | Assault by sharp object | 144 | 17 | 8.5 | (5.1 , 14) | | 0.57 |
| | S21 | Open wound of thorax | 71 | 17 | 4.2 | (2.5 , 7.1) | | 0.43 |
| | S11 | Open wound of neck | 30 | 10 | 3.0 | (1.5 , 6.1) | | 0.33 |
| | S41 | Open wound of shoulder and upper arm | 111 | 35 | 3.2 | (2.2 , 4.6) | | 0.31 |
| | T01 | Open wounds involving multiple body regions | 37 | 9 | 4.1 | (2 , 8.5) | | 0.29 |
| | S27 | Injury of other and unspecified intrathoracic organs | NR | <6 | 17.6 | (7.1 , 43.3) | | 0.27 |
| | X78 | Intentional self-harm by sharp object | 167 | 39 | 4.3 | (3 , 6.1) | | 0.27 |
| | S31 | Open wound of abdomen, lower back, pelvis and external genitals | 172 | 53 | 3.2 | (2.4 , 4.4) | | 0.26 |
| | S51 | Open wound of elbow and forearm | 669 | 183 | 3.7 | (3.1 , 4.3) | | 0.23 |
| 37 | | **Pharmacology Emergencies: Adverse Reactions to Antibiotics and Other Substances** | **3,851** | **1,870** | **2.2** | **(2 , 2.3)** | **<0.0001** | - |
| | Y40 | Systemic antibiotics | 316 | 154 | 2.1 | (1.7 , 2.5) | | 0.55 |
| | L27 | Dermatitis due to substances taken internally | 141 | 68 | 2.1 | (1.6 , 2.8) | | 0.49 |
| | T88 | Other complications of surgical and medical care, not elsewhere classified | 311 | 154 | 2.0 | (1.7 , 2.5) | | 0.42 |
| | T78 | Adverse effects, not elsewhere classified | 936 | 395 | 2.4 | (2.1 , 2.7) | | 0.40 |
| | Y57 | Other and unspecified drugs and medicaments | 171 | 66 | 2.6 | (2 , 3.5) | | 0.31 |
| | R21 | Rash and other nonspecific skin eruption | 1,307 | 810 | 1.6 | (1.5 , 1.8) | | 0.28 |
| | L50 | Urticaria | 615 | 345 | 1.8 | (1.6 , 2.1) | | 0.27 |
| | X58 | Exposure to other specified factors | 1,127 | 338 | 3.4 | (3 , 3.8) | | 0.24 |
| 38 | | **Trauma: Adult & Child Abuse and Sexual Assault** | **176** | **34** | **5.4** | **(3.7 , 7.9)** | **<0.0001** | - |
| | T74 | Adult and child abuse, neglect and other maltreatment, confirmed | 62 | 18 | 3.6 | (2.1 , 6.1) | | 0.79 |
| | Y05 | Sexual assault by bodily force | 62 | 18 | 3.4 | (2 , 5.8) | | 0.63 |
| | Y07 | Perpetrator of assault, maltreatment and neglect | 118 | 13 | 9.8 | (5.4 , 17.7) | | 0.57 |
| 39 | | **Environmental Exposures: Burn and Chemical Poisoning** | **436** | **127** | **3.5** | **(2.8 , 4.2)** | **<0.0001** | - |
| | T26 | Burn and corrosion confined to eye and adnexa | 100 | 31 | 3.2 | (2.2 , 4.8) | | 0.77 |
| | W89 | Exposure to man-made visible and ultraviolet light | 33 | 16 | 2.1 | (1.1 , 3.7) | | 0.66 |
| | X49 | Accidental poisoning by and exposure to other and unspecified chemicals and noxious substances | 279 | 83 | 3.4 | (2.7 , 4.3) | | 0.44 |
| | X08 | Exposure to other specified smoke, fire and flames | 60 | 11 | 5.5 | (2.9 , 10.4) | | 0.26 |
| | T54 | Toxic effect of corrosive substances | NR | <6 | 6.0 | (2.1 , 17.3) | | 0.21 |
| | T65 | Toxic effect of other and unspecified substances | 55 | 22 | 2.5 | (1.5 , 4.1) | | 0.21 |
| 40 | | **Gynecology: Infections and Other** | **571** | **273** | **2.1** | **(1.8 , 2.4)** | **<0.0001** | - |
| | N77 | Vulvovaginal ulceration and inflammation in diseases classified elsewhere | 134 | 56 | 2.4 | (1.8 , 3.3) | | 0.80 |
| | B37 | Candidiasis | 368 | 181 | 2.0 | (1.7 , 2.4) | | 0.66 |
| | A60 | Anogenital herpesviral [herpes simplex] infections | 55 | 15 | 3.7 | (2.1 , 6.5) | | 0.48 |
| | N76 | Other inflammation of vagina and vulva | 178 | 86 | 2.1 | (1.6 , 2.7) | | 0.28 |
| 41 | | **Pharmacology Emergencies: Poisoning due to Drugs Acting on Autonomic Nervous System** | **33** | **13** | **2.5** | **(1.3 , 4.8)** | **0.0044** | - |
| | X63 | Intentional self-poisoning by and exposure to other drugs acting on the autonomic nervous system | NR | <6 | 2.7 | (0.7 , 10.1) | | 0.83 |
| | T44 | Poisoning by, adverse effect of and underdosing of drugs primarily affecting the autonomic nervous system | 33 | 13 | 2.5 | (1.3 , 4.8) | | 0.82 |
| 42 | | **Environmental Exposures: Toxic Effect of Gases, Fumes and Vapours** | **159** | **46** | **3.5** | **(2.5 , 4.8)** | **<0.0001** | - |
| | T59 | Toxic effect of other gases, fumes and vapors | 93 | 28 | 3.3 | (2.2 , 5.1) | | 0.86 |
| | X47 | Accidental poisoning by and exposure to other gases and vapours | 67 | 20 | 3.4 | (2 , 5.5) | | 0.82 |
| | X09 | Exposure to unspecified smoke, fire and flames | 59 | 18 | 3.3 | (1.9 , 5.6) | | 0.24 |
| 43 | | **Environmental Exposures: Exposure to electrical Current** | **64** | **19** | **3.4** | **(2 , 5.6)** | **<0.0001** | - |

| | | | | | | |
|---|---|---|---|---|---|---|
| | W87 Exposure to unspecified electric current | 32 | 9 | 3.6 | (1.7 , 7.4) | 0.87 |
| | T75 Other and unspecified effects of other external causes | 60 | 17 | 3.5 | (2.1 , 6) | 0.87 |

NR - Not reported due to small cell size

TBI - Traumatic Brain Injury

**Supplementary Table 2 - ICD-10 Codes not included in the Factors.** Provides all ICD-10 codes not included in any factor (in alphabetical order), including their descriptions, frequencies (in TBI and reference population), and odds ratio (and 95% confidence interval) from multiple testing results.

| ICD-10 Codes | Description | Frequency | | Odds Ratio | Confidence Interval |
|---|---|---|---|---|---|
| | | TBI | Reference Population | | |
| **I. Certain infectious and parasitic diseases (A00-B99)** | | | | | |
| A04 | Other bacterial intestinal infections | 150 | 57 | 2.6 | (1.9 , 3.6) |
| A08 | Viral and other specified intestinal infections | 600 | 314 | 2.0 | (1.7 , 2.3) |
| A09 | Other gastroenteritis and colitis of infectious and unspecified origin | 2,028 | 633 | 3.3 | (3 , 3.6) |
| A37 | Whooping cough | 17 | 10 | 1.7 | (0.8 , 3.7) |
| A38 | Scarlet fever | 60 | 26 | 2.3 | (1.5 , 3.7) |
| A46 | Erysipelas | 19 | 8 | 2.4 | (1 , 5.4) |
| A49 | Bacterial infection of unspecified site | 96 | 39 | 2.5 | (1.7 , 3.6) |
| A64 | Unspecified sexually transmitted disease | 45 | 6 | 7.5 | (3.2 , 17.6) |
| A74 | Other diseases caused by chlamydiae | NR | <6 | 3.5 | (1.2 , 10.6) |
| B00 | Herpesviral [herpes simplex] infections | 154 | 79 | 2.0 | (1.5 , 2.6) |
| B01 | Varicella [chickenpox] | 119 | 89 | 1.3 | (1 , 1.8) |
| B02 | Zoster [herpes zoster] | 315 | 181 | 1.7 | (1.5 , 2.1) |
| B07 | Viral warts | 147 | 88 | 1.7 | (1.3 , 2.2) |
| B08 | Other viral infections characterized by skin and mucous membrane lesions, not elsewhere classified | 131 | 51 | 2.6 | (1.9 , 3.5) |
| B09 | Unspecified viral infection characterized by skin and mucous membrane lesions | 161 | 71 | 2.3 | (1.7 , 3) |
| B27 | Infectious mononucleosis | 265 | 112 | 2.4 | (1.9 , 3) |
| B35 | Dermatophytosis | 170 | 65 | 2.6 | (2 , 3.5) |
| B80 | Enterobiasis | 35 | 12 | 2.9 | (1.5 , 5.6) |
| B86 | Scabies | 57 | 22 | 2.6 | (1.6 , 4.2) |
| B97 | Viral agents as the cause of diseases classified elsewhere | 199 | 84 | 2.4 | (1.8 , 3.1) |
| **II. Neoplasms (C00-D48)** | | | | | |
| C90 | Multiple myeloma and malignant plasma cell neoplasms | 31 | 12 | 2.6 | (1.3 , 5) |
| **IV. Endocrine, nutritional and metabolic diseases (E00-E90)** | | | | | |
| E03 | Other hypothyroidism | 234 | 130 | 1.8 | (1.5 , 2.3) |
| E16 | Other disorders of pancreatic internal secretion | 95 | 39 | 2.4 | (1.7 , 3.5) |
| E22 | Hyperfunction of pituitary gland | 25 | 15 | 1.7 | (0.9 , 3.2) |
| E46 | Unspecified protein-energy malnutrition | 41 | 16 | 2.6 | (1.4 , 4.6) |
| **V. Mental and behavioural disorders (F00-F99)** | | | | | |
| F01 | Vascular dementia | 49 | 17 | 2.9 | (1.7 , 5) |
| F06 | Other mental disorders due to known physiological condition | 75 | 18 | 4.2 | (2.5 , 7) |
| F07 | Personality and behavioral disorders due to known physiological condition | NR | <6 | 56.7 | (18.1 , 177.5) |
| F15 | Other stimulant related disorders | 40 | 18 | 2.2 | (1.3 , 3.9) |
| F45 | Somatoform disorders | 71 | 22 | 3.2 | (2 , 5.2) |
| F48 | Other nonpsychotic mental disorders | 26 | 13 | 2.0 | (1 , 3.9) |
| F51 | Sleep disorders not due to a substance or known physiological condition | 30 | 7 | 4.3 | (1.9 , 9.8) |
| **VI. Diseases of the nervous system (G00-G99)** | | | | | |
| G03 | Meningitis due to other and unspecified causes | 28 | 9 | 3.1 | (1.5 , 6.6) |

| | | | | | |
|---|---|---|---|---|---|
| G06 | Intracranial and intraspinal abscess and granuloma | 11 | 6 | 1.8 | (0.7 , 5) |
| G25 | Other extrapyramidal and movement disorders | 56 | 22 | 2.5 | (1.6 , 4.2) |
| G31 | Other degenerative diseases of nervous system, not elsewhere classified | 33 | 6 | 5.5 | (2.3 , 13.1) |
| G35 | Multiple sclerosis | 44 | 18 | 2.4 | (1.4 , 4.2) |
| G44 | Other headache syndromes | 272 | 78 | 3.5 | (2.7 , 4.5) |
| G47 | Sleep disorders | 319 | 186 | 1.7 | (1.4 , 2.1) |
| G50 | Disorders of trigeminal nerve | 57 | 20 | 2.9 | (1.7 , 4.7) |
| G62 | Other and unspecified polyneuropathies | 80 | 23 | 3.5 | (2.2 , 5.5) |
| G91 | Hydrocephalus | 58 | 19 | 3.1 | (1.8 , 5.1) |
| **VII. Diseases of the eye and adnexa (H00-H59)** | | | | | |
| H00 | Hordeolum and chalazion | 201 | 126 | 1.6 | (1.3 , 2) |
| H05 | Disorders of orbit | 70 | 37 | 1.9 | (1.3 , 2.8) |
| H11 | Other disorders of conjunctiva | 206 | 115 | 1.8 | (1.4 , 2.3) |
| H16 | Keratitis | 156 | 70 | 2.2 | (1.7 , 3) |
| H20 | Iridocyclitis | 46 | 22 | 2.1 | (1.3 , 3.5) |
| H53 | Visual disturbances | 307 | 170 | 1.8 | (1.5 , 2.2) |
| H57 | Other disorders of eye and adnexa | 689 | 451 | 1.5 | (1.4 , 1.7) |
| **VIII. Diseases of the ear and mastoid process (H60-H95)** | | | | | |
| H61 | Other disorders of external ear | 315 | 206 | 1.5 | (1.3 , 1.8) |
| H72 | Perforation of tympanic membrane | 123 | 64 | 2.0 | (1.4 , 2.7) |
| H81 | Disorders of vestibular function | 326 | 210 | 1.6 | (1.3 , 1.9) |
| H83 | Other diseases of inner ear | 154 | 83 | 1.9 | (1.4 , 2.4) |
| **IX. Diseases of the circulatory system (I00-I99)** | | | | | |
| I26 | Pulmonary embolism | 249 | 108 | 2.3 | (1.9 , 2.9) |
| I27 | Other pulmonary heart diseases | 69 | 29 | 2.4 | (1.5 , 3.7) |
| I31 | Other diseases of pericardium | 71 | 48 | 1.5 | (1 , 2.1) |
| I47 | Paroxysmal tachycardia | 259 | 147 | 1.8 | (1.4 , 2.2) |
| I49 | Other cardiac arrhythmias | 280 | 178 | 1.6 | (1.3 , 1.9) |
| I60 | Nontraumatic subarachnoid hemorrhage | 52 | 9 | 5.8 | (2.8 , 11.7) |
| I87 | Other disorders of veins | 43 | 20 | 2.2 | (1.3 , 3.7) |
| I88 | Nonspecific lymphadenitis | 218 | 77 | 2.8 | (2.2 , 3.7) |
| I95 | Hypotension | 447 | 205 | 2.2 | (1.9 , 2.6) |
| **X. Diseases of the respiratory system (J00-J99)** | | | | | |
| J01 | Acute sinusitis | 509 | 252 | 2.0 | (1.7 , 2.4) |
| J04 | Acute laryngitis and tracheitis | 117 | 62 | 1.9 | (1.4 , 2.6) |
| J11 | Influenza due to unidentified influenza virus | 731 | 236 | 3.1 | (2.7 , 3.6) |
| J21 | Acute bronchiolitis | 182 | 128 | 1.4 | (1.1 , 1.8) |
| J22 | Unspecified acute lower respiratory infection | 323 | 132 | 2.5 | (2 , 3.1) |
| J30 | Vasomotor and allergic rhinitis | 96 | 59 | 1.6 | (1.2 , 2.3) |
| J32 | Chronic sinusitis | 574 | 288 | 2.0 | (1.7 , 2.3) |
| J36 | Peritonsillar abscess | 115 | 60 | 1.9 | (1.4 , 2.6) |
| J39 | Other diseases of upper respiratory tract | 45 | 42 | 1.1 | (0.7 , 1.6) |
| J81 | Pulmonary edema | 79 | 53 | 1.5 | (1.1 , 2.1) |
| J93 | Pneumothorax and air leak | 75 | 28 | 2.7 | (1.7 , 4.1) |
| **XI. Diseases of the digestive system (K00-K93)** | | | | | |
| K04 | Diseases of pulp and periapical tissues | 617 | 258 | 2.4 | (2.1 , 2.8) |
| K06 | Other disorders of gingiva and edentulous alveolar ridge | 21 | 15 | 1.4 | (0.7 , 2.7) |
| K08 | Other disorders of teeth and supporting structures | 530 | 233 | 2.3 | (2 , 2.7) |
| K10 | Other diseases of jaws | 101 | 52 | 1.9 | (1.4 , 2.7) |
| K11 | Diseases of salivary glands | 84 | 49 | 1.7 | (1.2 , 2.4) |
| K12 | Stomatitis and related lesions | 181 | 102 | 1.8 | (1.4 , 2.3) |
| K13 | Other diseases of lip and oral mucosa | 118 | 64 | 1.8 | (1.4 , 2.5) |

| | | | | | |
|---|---|---|---|---|---|
| K27 | Peptic ulcer, site unspecified | 140 | 60 | 2.3 | (1.7 , 3.2) |
| K46 | Unspecified abdominal hernia | 68 | 24 | 2.8 | (1.8 , 4.5) |
| K59 | Other functional intestinal disorders | 1,725 | 800 | 2.2 | (2 , 2.4) |
| K64 | Hemorrhoids and perianal venous thrombosis | 175 | 62 | 2.9 | (2.1 , 3.8) |
| K85 | Acute pancreatitis | 245 | 122 | 2.0 | (1.6 , 2.5) |
| K86 | Other diseases of pancreas | 69 | 25 | 2.8 | (1.7 , 4.4) |
| **XII. Diseases of the skin and subcutaneous tissue (L00-L99)** | | | | | |
| L01 | Impetigo | 314 | 134 | 2.4 | (1.9 , 2.9) |
| L04 | Acute lymphadenitis | 71 | 29 | 2.4 | (1.6 , 3.8) |
| L23 | Allergic contact dermatitis | 316 | 189 | 1.7 | (1.4 , 2) |
| L25 | Unspecified contact dermatitis | 351 | 123 | 2.9 | (2.3 , 3.5) |
| L29 | Pruritus | 116 | 70 | 1.7 | (1.2 , 2.3) |
| L30 | Other and unspecified dermatitis | 365 | 211 | 1.7 | (1.5 , 2.1) |
| L42 | Pityriasis rosea | 31 | 18 | 1.7 | (1 , 3.1) |
| L55 | Sunburn | 72 | 38 | 1.9 | (1.3 , 2.8) |
| L60 | Nail disorders | 233 | 157 | 1.5 | (1.2 , 1.8) |
| L72 | Follicular cysts of skin and subcutaneous tissue | 256 | 192 | 1.3 | (1.1 , 1.6) |
| L73 | Other follicular disorders | 149 | 68 | 2.2 | (1.7 , 2.9) |
| L98 | Other disorders of skin and subcutaneous tissue, not elsewhere classified | 259 | 116 | 2.2 | (1.8 , 2.8) |
| **XIII. Diseases of the musculoskeletal system and connective tissue (M00-M99)** | | | | | |
| M00 | Pyogenic arthritis | 48 | 35 | 1.4 | (0.9 , 2.1) |
| M06 | Other rheumatoid arthritis | 72 | 46 | 1.6 | (1.1 , 2.3) |
| M10 | Gout | 312 | 186 | 1.7 | (1.4 , 2) |
| M13 | Other arthritis | 250 | 143 | 1.7 | (1.4 , 2.1) |
| M19 | Other and unspecified osteoarthritis | 231 | 144 | 1.6 | (1.3 , 2) |
| M43 | Other deforming dorsopathies | 216 | 107 | 2.0 | (1.6 , 2.5) |
| M51 | Thoracic, thoracolumbar, and lumbosacral intervertebral disc disorders | 260 | 157 | 1.7 | (1.4 , 2) |
| M53 | Other and unspecified dorsopathies, not elsewhere classified | 40 | 20 | 2.0 | (1.2 , 3.4) |
| M60 | Myositis | 38 | 20 | 1.9 | (1.1 , 3.3) |
| M62 | Other disorders of muscle | 775 | 268 | 2.9 | (2.5 , 3.4) |
| M65 | Synovitis and tenosynovitis | 271 | 183 | 1.5 | (1.2 , 1.8) |
| M70 | Soft tissue disorders related to use, overuse and pressure | 331 | 126 | 2.7 | (2.2 , 3.3) |
| M71 | Other bursopathies | 123 | 61 | 2.0 | (1.5 , 2.7) |
| M72 | Fibroblastic disorders | 161 | 78 | 2.1 | (1.6 , 2.7) |
| M75 | Shoulder lesions | 395 | 251 | 1.6 | (1.3 , 1.9) |
| M76 | Enthesopathies, lower limb, excluding foot | 95 | 30 | 3.2 | (2.1 , 4.8) |
| M77 | Other enthesopathies | 301 | 96 | 3.2 | (2.5 , 4) |
| M80 | Osteoporosis with current pathological fracture | 34 | 7 | 4.9 | (2.2 , 11) |
| M84 | Disorder of continuity of bone | 72 | 50 | 1.4 | (1 , 2.1) |
| M92 | Other juvenile osteochondrosis | 49 | 17 | 2.9 | (1.7 , 5) |
| M94 | Other disorders of cartilage | 293 | 99 | 3.0 | (2.4 , 3.7) |
| **XIV. Diseases of the genitourinary system (N00-N99)** | | | | | |
| N45 | Orchitis and epididymitis | 189 | 85 | 2.3 | (1.7 , 2.9) |
| N48 | Other disorders of penis | 192 | 103 | 1.9 | (1.5 , 2.4) |
| N50 | Other and unspecified disorders of male genital organs | 226 | 133 | 1.7 | (1.4 , 2.1) |
| N61 | Inflammatory disorders of breast | 72 | 35 | 2.1 | (1.4 , 3.2) |
| N64 | Other disorders of breast | 72 | 36 | 2.0 | (1.3 , 3) |
| N71 | Inflammatory disease of uterus, except cervix | 14 | 7 | 2.0 | (0.8 , 5) |

| | | | | | |
|---|---|---|---|---|---|
| **XVIII. Symptoms, signs and abnormal clinical and laboratory findings, not elswhere classified (R00-R99)** | | | | | |
| R00 | Abnormalities of heart beat | 994 | 567 | 1.8 | (1.6 , 2) |
| R03 | Abnormal blood-pressure reading, without diagnosis | 43 | 33 | 1.3 | (0.8 , 2.1) |
| R04 | Hemorrhage from respiratory passages | 710 | 304 | 2.4 | (2.1 , 2.7) |
| R09 | Other symptoms and signs involving the circulatory and respiratory system | 191 | 73 | 2.6 | (2 , 3.4) |
| R13 | Aphagia and dysphagia | 322 | 203 | 1.6 | (1.3 , 1.9) |
| R20 | Disturbances of skin sensation | 606 | 299 | 2.0 | (1.8 , 2.4) |
| R23 | Other skin changes | 151 | 84 | 1.8 | (1.4 , 2.3) |
| R25 | Abnormal involuntary movements | 434 | 165 | 2.7 | (2.2 , 3.2) |
| R27 | Other lack of coordination | 78 | 16 | 4.9 | (2.8 , 8.3) |
| R30 | Pain associated with micturition | 313 | 219 | 1.4 | (1.2 , 1.7) |
| R32 | Unspecified urinary incontinence | 77 | 74 | 1.0 | (0.8 , 1.4) |
| R40 | Somnolence, stupor and coma | 130 | 40 | 3.3 | (2.3 , 4.6) |
| R42 | Dizziness and giddiness | 1,615 | 802 | 2.1 | (1.9 , 2.3) |
| R51 | Headache | 2,336 | 902 | 2.7 | (2.5 , 2.9) |
| R52 | Pain, not elsewhere classified | 709 | 173 | 4.2 | (3.5 , 4.9) |
| R55 | Syncope and collapse | 1,728 | 682 | 2.6 | (2.4 , 2.8) |
| R59 | Enlarged lymph nodes | 242 | 113 | 2.2 | (1.7 , 2.7) |
| R60 | Edema, not elsewhere classified | 376 | 153 | 2.5 | (2 , 3) |
| R63 | Symptoms and signs concerning food and fluid intake | 239 | 149 | 1.6 | (1.3 , 2) |
| R68 | Other general symptoms and signs | 321 | 157 | 2.1 | (1.7 , 2.5) |
| R74 | Abnormal serum enzyme levels | 87 | 35 | 2.5 | (1.7 , 3.7) |
| R79 | Other abnormal findings of blood chemistry | 101 | 63 | 1.6 | (1.2 , 2.2) |
| **XIX. Injury, poisoning and certain other consequences of external causes (S00-T98)** | | | | | |
| S16 | Injury of muscle, fascia and tendon at neck level | 77 | 17 | 4.5 | (2.7 , 7.7) |
| S29 | Other and unspecified injuries of thorax | 457 | 109 | 4.2 | (3.4 , 5.2) |
| S36 | Injury of intra-abdominal organs | 72 | 19 | 3.8 | (2.3 , 6.3) |
| S37 | Injury of urinary and pelvic organs | 46 | 18 | 2.6 | (1.5 , 4.4) |
| S42 | Fracture of shoulder and upper arm | 918 | 365 | 2.6 | (2.3 , 2.9) |
| S43 | Dislocation and sprain of joints and ligaments of shoulder girdle | 1,033 | 313 | 3.3 | (2.9 , 3.8) |
| S46 | Injury of muscle, fascia and tendon at shoulder and upper arm level | 329 | 93 | 3.6 | (2.8 , 4.5) |
| S53 | Dislocation and sprain of joints and ligaments of elbow | 271 | 109 | 2.5 | (2 , 3.1) |
| S54 | Injury of nerves at forearm level | 22 | 7 | 3.1 | (1.3 , 7.4) |
| S56 | Injury of muscle, fascia and tendon at forearm level | 92 | 25 | 3.7 | (2.4 , 5.7) |
| S66 | Injury of muscle, fascia and tendon at wrist and hand level | 156 | 51 | 3.1 | (2.2 , 4.2) |
| S67 | Crushing injury of wrist, hand and fingers | 216 | 73 | 3.0 | (2.3 , 3.9) |
| S70 | Superficial injury of hip and thigh | 552 | 147 | 3.8 | (3.1 , 4.5) |
| S71 | Open wound of hip and thigh | 178 | 69 | 2.6 | (2 , 3.4) |
| S73 | Dislocation and sprain of joint and ligaments of hip | 75 | 41 | 1.8 | (1.3 , 2.7) |
| S76 | Injury of muscle, fascia and tendon at hip and thigh level | 116 | 44 | 2.6 | (1.9 , 3.7) |
| S79 | Other and unspecified injuries of hip and thigh | 245 | 75 | 3.3 | (2.5 , 4.2) |
| S80 | Superficial injury of knee and lower leg | 1,792 | 521 | 3.5 | (3.2 , 3.9) |
| S81 | Open wound of knee and lower leg | 824 | 302 | 2.8 | (2.4 , 3.1) |
| S86 | Injury of muscle, fascia and tendon at lower leg level | 139 | 49 | 2.8 | (2 , 3.9) |
| S96 | Injury of muscle and tendon at ankle and foot level | 52 | 14 | 3.7 | (2.1 , 6.7) |
| S97 | Crushing injury of ankle and foot | 29 | 13 | 2.2 | (1.2 , 4.3) |
| T03 | Dislocations, sprains and strains involving multiple body regions | 49 | 21 | 2.3 | (1.4 , 3.9) |

| | | | | | |
|---|---|---|---|---|---|
| T06 | Other injuries involving multiple body regions, not elsewhere classified | 82 | 19 | 4.3 | (2.6 , 7.1) |
| T07 | Unspecified multiple injuries | 57 | 17 | 3.4 | (2 , 5.8) |
| T09 | Other injuries of spine and trunk, level unspecified | 411 | 106 | 3.9 | (3.2 , 4.9) |
| T11 | Other injuries of upper limb, level unspecified | 334 | 127 | 2.6 | (2.1 , 3.2) |
| T13 | Other injuries of lower limb, level unspecified | 303 | 113 | 2.7 | (2.2 , 3.3) |
| T19 | Foreign body in genitourinary tract | 39 | 21 | 1.9 | (1.1 , 3.2) |
| T52 | Toxic effect of organic solvents | 33 | 12 | 2.8 | (1.4 , 5.3) |
| T79 | Certain early complications of trauma, not elsewhere classified | 101 | 25 | 4.0 | (2.6 , 6.3) |
| T80 | Complications following infusion, transfusion and therapeutic injection | 55 | 30 | 1.8 | (1.2 , 2.9) |
| **XX. External causes of morbidity and mortality (V01-Y98)** | | | | | |
| V03 | Pedestrian injured in collision with car, pick-up truck or van | 216 | 57 | 3.8 | (2.8 , 5.1) |
| V09 | Pedestrian injured in other and unspecified transport accidents | 24 | 8 | 3.0 | (1.3 , 6.7) |
| V13 | Pedal cycle rider injured in collision with car, pick-up truck or van | 117 | 30 | 3.9 | (2.6 , 5.8) |
| V17 | Pedal cycle rider injured in collision with fixed or stationary object | 35 | 6 | 5.8 | (2.5 , 13.9) |
| V19 | Pedal cycle rider injured in other and unspecified transport accidents | 145 | 52 | 2.8 | (2.1 , 3.9) |
| V23 | Motorcycle rider injured in collision with car, pick-up truck or van | 21 | 7 | 3.0 | (1.3 , 7.1) |
| V28 | Motorcycle rider injured in noncollision transport accident | 174 | 48 | 3.7 | (2.7 , 5.1) |
| V29 | Motorcycle rider injured in other and unspecified transport accidents | 58 | 18 | 3.2 | (1.9 , 5.5) |
| V44 | Car occupant injured in collision with heavy transport vehicle or bus | 28 | 12 | 2.3 | (1.2 , 4.6) |
| V47 | Car occupant injured in collision with fixed or stationary object | 146 | 33 | 4.4 | (3 , 6.5) |
| V48 | Car occupant injured in noncollision transport accident | 256 | 66 | 3.9 | (3 , 5.1) |
| V49 | Car occupant injured in other and unspecified transport accidents | 175 | 50 | 3.5 | (2.6 , 4.8) |
| V53 | Occupant of pick-up truck or van injured in collision with car, pick-up truck or van | 43 | 6 | 7.2 | (3.1 , 16.8) |
| V58 | Occupant of pick-up truck or van injured in noncollision transport accident | 58 | 16 | 3.6 | (2.1 , 6.3) |
| V73 | Bus occupant injured in collision with car, pick-up truck or van | NR | <6 | 5.0 | (1.1 , 22.8) |
| V78 | Bus occupant injured in noncollision transport accident | 53 | 16 | 3.3 | (1.9 , 5.8) |
| V80 | Animal-rider or occupant of animal-drawn vehicle injured in transport accident | 181 | 32 | 5.7 | (3.9 , 8.2) |
| V86 | Occupant of special all-terrain or other off-road motor vehicle, injured in transport accident | 418 | 124 | 3.4 | (2.8 , 4.2) |
| V94 | Other and unspecified water transport accidents | 38 | 6 | 6.3 | (2.7 , 15) |
| W00 | Fall due to ice and snow | 1,013 | 276 | 3.7 | (3.3 , 4.3) |
| W03 | Other fall on same level due to collision with another person | 135 | 24 | 5.8 | (3.7 , 9.1) |
| W04 | Fall while being carried or supported by other persons | 42 | 11 | 3.8 | (2 , 7.4) |
| W07 | Fall from chair | 267 | 60 | 4.5 | (3.4 , 5.9) |
| W08 | Fall from other furniture | 197 | 40 | 5.0 | (3.6 , 7.1) |
| W09 | Fall on and from playground equipment | 525 | 164 | 3.2 | (2.7 , 3.9) |

| | | | | | |
|---|---|---|---|---|---|
| W10 | Fall on and from stairs and steps | 1,917 | 521 | 3.8 | (3.4 , 4.1) |
| W11 | Fall on and from ladder | 243 | 78 | 3.1 | (2.4 , 4) |
| W13 | Fall from, out of or through building or structure | 113 | 29 | 3.9 | (2.6 , 5.9) |
| W14 | Fall from tree | 67 | 26 | 2.6 | (1.6 , 4.1) |
| W16 | Fall, jump or diving into water | 66 | 13 | 5.4 | (2.9 , 10) |
| W17 | Other fall from one level to another | 758 | 210 | 3.7 | (3.1 , 4.3) |
| W20 | Struck by thrown, projected or falling object | 1,009 | 252 | 4.2 | (3.6 , 4.8) |
| W46 | Contact with hypodermic needle | 69 | 28 | 2.5 | (1.6 , 3.8) |
| W50 | Accidental hit, strike, kick, twist, bite or scratch by another person | 926 | 158 | 6.0 | (5.1 , 7.1) |
| W54 | Contact with dog | 503 | 138 | 3.7 | (3 , 4.5) |
| W55 | Contact with other mammals | 397 | 109 | 3.7 | (3 , 4.5) |
| W57 | Bitten or stung by nonvenomous insect and other nonvenomous arthropods | 506 | 169 | 3.0 | (2.5 , 3.6) |
| W64 | Exposure to other animate mechanical forces | 22 | 7 | 3.1 | (1.3 , 7.4) |
| W79 | Inhalation and ingestion of food causing obstruction of respiratory tract | 12 | 15 | 0.8 | (0.4 , 1.7) |
| X13 | Contact with steam and other hot vapors | 14 | 7 | 2.0 | (0.8 , 5) |
| X83 | Intentional self-harm by other specified means | NR | <6 | 2.2 | (0.8 , 6.3) |
| X84 | Intentional self-harm by unspecified means | NR | <6 | 3.8 | (1.4 , 10.2) |
| Y00 | Assault by blunt object | 132 | 6 | 22.0 | (9.7 , 49.9) |
| Y08 | Assault by other specified means | 91 | 15 | 6.1 | (3.5 , 10.5) |
| Y28 | Contact with sharp object, undetermined intent | 81 | 22 | 3.7 | (2.3 , 5.9) |
| Y29 | Contact with blunt object, undetermined intent | 16 | 6 | 2.7 | (1 , 6.8) |
| Y34 | Unspecified event, undetermined intent | 32 | 9 | 3.6 | (1.7 , 7.4) |
| Y35 | Legal intervention | 51 | 7 | 7.3 | (3.3 , 16.1) |
| Y42 | Hormones and their synthetic substitutes and antagonists, not elsewhere classified | 59 | 35 | 1.7 | (1.1 , 2.6) |
| Y45 | Analgesics, antipyretics and anti-inflammatory drugs | 230 | 94 | 2.5 | (1.9 , 3.1) |
| Y47 | Sedatives, hypnotics and antianxiety drugs | NR | <6 | 9.0 | (2.7 , 29.7) |
| Y49 | Psychotropic drugs, not elsewhere classified | 101 | 33 | 3.1 | (2.1 , 4.5) |
| Y51 | Drugs primarily affecting the autonomic nervous system | 54 | 37 | 1.5 | (1 , 2.2) |
| Y52 | Agents primarily affecting the cardiovascular system | 98 | 62 | 1.6 | (1.2 , 2.2) |
| Y54 | Agents primarily affecting water-balance and mineral and uric acid metabolism | 70 | 21 | 3.6 | (2.2 , 6) |
| Y85 | Sequelae of transport accidents | 25 | 9 | 2.8 | (1.3 , 6) |
| Y86 | Sequelae of other accidents | 58 | 19 | 3.1 | (1.8 , 5.1) |
| Y89 | Sequelae of other external causes | 23 | 8 | 2.9 | (1.3 , 6.4) |
| Y91 | Evidence of alcohol involvement determined by level of intoxication | NR | <6 | 60.0 | (14.8 , 242.7) |
| **XXI. Factors influencing health status and contact with health services (Z00-Z99)** | | | | | |
| Z00 | Encounter for general examination without complaint, suspected or reported diagnosis | 161 | 56 | 2.9 | (2.1 , 4) |
| Z01 | Encounter for other special examination without complaint, suspected or reported diagnosis | 896 | 446 | 2.1 | (1.8 , 2.3) |
| Z02 | Encounter for administrative examination | 361 | 106 | 3.5 | (2.8 , 4.3) |
| Z03 | Encounter for medical observation for suspected diseases and conditions ruled out | 585 | 464 | 1.3 | (1.1 , 1.4) |
| Z04 | Encounter for examination and observation for other reasons | 987 | 342 | 2.9 | (2.6 , 3.3) |
| Z11 | Encounter for screening for infectious and parasitic diseases | 153 | 52 | 2.9 | (2.1 , 4) |

| | | | | | |
|---|---|---|---|---|---|
| Z20 | Contact with and (suspected) exposure to communicable diseases | 100 | 37 | 2.7 | (1.9 , 3.9) |
| Z23 | Need for immunization against single bacterial diseases | 152 | 73 | 2.1 | (1.6 , 2.8) |
| Z27 | Need for immunization against combinations of infectious diseases | 78 | 28 | 2.9 | (1.8 , 4.4) |
| Z45 | Encounter for adjustment and management of implanted device | 176 | 132 | 1.3 | (1.1 , 1.7) |
| Z54 | Convalescence | 227 | 159 | 1.4 | (1.2 , 1.8) |
| Z64 | Problems related to certain psychosocial circumstances | NR | <6 | 6.5 | (2.3 , 18.6) |
| Z86 | Personal history of certain other diseases | 727 | 491 | 1.5 | (1.3 , 1.7) |
| Z96 | Presence of other functional implants | 183 | 121 | 1.5 | (1.2 , 1.9) |
| Z97 | Presence of other devices | 34 | 9 | 3.8 | (1.8 , 7.9) |

NR - Not Reported due to small cell size

TBI - Traumatic Brain Injury

**Supplementary Table 3 - Scree Table.** Provides eigenvalues and their differences used in preparation of scree plots, as well as the proportion/cumulative proportion of total variance explained by each factor; used to make a decision on the number of factors to include in the final model.

| Factor Number | Eigenvalue | Eigenvalue Difference | Proportion of Variance | Cumulative Proportion of Variance |
|---|---|---|---|---|
| 1 | 9.951 | 3.218 | 0.017 | 0.017 |
| 2 | 6.733 | 2.135 | 0.012 | 0.029 |
| 3 | 4.598 | 1.622 | 0.008 | 0.037 |
| 4 | 2.976 | 0.155 | 0.005 | 0.042 |
| 5 | 2.822 | 0.048 | 0.005 | 0.047 |
| 6 | 2.774 | 0.053 | 0.005 | 0.051 |
| 7 | 2.721 | 0.160 | 0.005 | 0.056 |
| 8 | 2.561 | 0.143 | 0.004 | 0.060 |
| 9 | 2.418 | 0.123 | 0.004 | 0.065 |
| 10 | 2.295 | 0.072 | 0.004 | 0.069 |
| 11 | 2.223 | 0.119 | 0.004 | 0.072 |
| 12 | 2.104 | 0.062 | 0.004 | 0.076 |
| 13 | 2.041 | 0.028 | 0.004 | 0.079 |
| 14 | 2.014 | 0.035 | 0.004 | 0.083 |
| 15 | 1.979 | 0.048 | 0.003 | 0.086 |
| 16 | 1.931 | 0.052 | 0.003 | 0.090 |
| 17 | 1.879 | 0.036 | 0.003 | 0.093 |
| 18 | 1.843 | 0.017 | 0.003 | 0.096 |
| 19 | 1.826 | 0.002 | 0.003 | 0.099 |
| 20 | 1.823 | 0.014 | 0.003 | 0.102 |
| 21 | 1.809 | 0.019 | 0.003 | 0.105 |
| 22 | 1.790 | 0.017 | 0.003 | 0.108 |
| 23 | 1.773 | 0.016 | 0.003 | 0.112 |
| 24 | 1.757 | 0.027 | 0.003 | 0.115 |
| 25 | 1.730 | 0.011 | 0.003 | 0.118 |
| 26 | 1.719 | 0.021 | 0.003 | 0.120 |
| 27 | 1.698 | 0.054 | 0.003 | 0.123 |
| 28 | 1.644 | 0.012 | 0.003 | 0.126 |
| 29 | 1.632 | 0.008 | 0.003 | 0.129 |
| 30 | 1.624 | 0.006 | 0.003 | 0.132 |
| 31 | 1.618 | 0.037 | 0.003 | 0.135 |
| 32 | 1.581 | 0.013 | 0.003 | 0.137 |
| 33 | 1.569 | 0.007 | 0.003 | 0.140 |
| 34 | 1.562 | 0.023 | 0.003 | 0.143 |
| 35 | 1.539 | 0.005 | 0.003 | 0.145 |
| 36 | 1.534 | 0.009 | 0.003 | 0.148 |
| 37 | 1.525 | 0.015 | 0.003 | 0.151 |
| 38 | 1.510 | 0.016 | 0.003 | 0.153 |
| 39 | 1.494 | 0.018 | 0.003 | 0.156 |

| | | | | |
|---|---|---|---|---|
| **40** | 1.476 | 0.011 | 0.003 | 0.158 |
| **41** | 1.464 | 0.008 | 0.003 | 0.161 |
| **42** | 1.456 | 0.011 | 0.003 | 0.163 |
| ***43*** | ***1.445*** | ***0.018*** | ***0.003*** | ***0.166*** |
| **44** | 1.427 | 0.015 | 0.003 | 0.168 |
| **45** | 1.412 | 0.017 | 0.002 | 0.171 |
| **46** | 1.395 | 0.007 | 0.002 | 0.173 |
| **47** | 1.388 | 0.006 | 0.002 | 0.175 |
| **48** | 1.382 | 0.020 | 0.002 | 0.178 |
| **49** | 1.363 | 0.004 | 0.002 | 0.180 |
| **50** | 1.359 | 0.014 | 0.002 | 0.183 |
